# LncRNA HITT stabilizes SIRT6 to restrain H3/H4 acetylation and suppress colorectal cancer growth

**DOI:** 10.1038/s41419-026-09107-z

**Published:** 2026-07-27

**Authors:** Bolun Li, Zhiyuan Xiang, Hao Liu, Qiannan Cheng, Haoyu Wang, Kaiting Chen, Jing Hu, Ying Hu

**Affiliations:** 1 Zhengzhou Advanced Research Institute, Harbin Institute of Technology, Zhengzhou, Henan Province China; 2 School of Life Science and Technology, Harbin Institute of Technology, Harbin, Heilongjiang Province China; 3 Key Laboratory of Science and Technology and Intelligent Equipment for the Prevention and Control of Major Chronic Diseases, Ministry of Industry and Information Technology, Harbin Institute of Technology, Harbin, Heilongjiang Province China

**Keywords:** Colon cancer, Targeted therapies

## Abstract

Histone post-translational modifications are intimately linked to human diseases, including cancers. While diverse regulatory mechanisms have been described, how long non-coding RNAs (lncRNAs) control histone acetylation remains incompletely understood. Here, integrative bioinformatics analysis implicates the lncRNA HITT (HIF-1α inhibitor at the translation level) in histone modification-related pathways. Mechanistically, HITT selectively suppresses H3/H4 acetylation in colorectal cancer cells. Using siRNA-based loss-of-function screening and pharmacological inhibition, we identify the sirtuin deacetylase SIRT6 as an essential mediator of this effect. HITT directly binds SIRT6 and increases SIRT6 protein abundance by attenuating its association with the E3 ubiquitin ligase mouse double minute 2 homolog (MDM2), thereby reducing MDM2-dependent ubiquitination and proteasomal turnover of SIRT6. Conversely, the SIRT6 agonist MDL-800 upregulates the transcription factor E2F1, thereby promoting E2F1-dependent transcription of HITT. Functionally, increased HITT expression cooperates with MDL-800 to inhibit colorectal cancer cell proliferation in vitro and suppress tumor growth in vivo. Collectively, these findings define a feed-forward HITT-SIRT6 axis that stabilizes SIRT6 and constrains histone H3/H4 acetylation homeostasis, highlighting a potential combination strategy for colorectal cancer therapy.

## Introduction

Eukaryotic DNA is packaged into chromatin by wrapping around histone octamers to form nucleosomes [1]. Post-translational modifications (PTMs) on histone tails constitute a central layer of epigenetic regulation, tuning chromatin accessibility and gene expression without altering DNA sequence [2]. These covalent modifications*—*including methylation [3], acetylation [4], phosphorylation [5], and ubiquitination [6]*—*are interpreted by dedicated “reader” proteins that recruit chromatin-modifying complexes and transcriptional machinery to shape transcriptional outcomes [7]. Among them, histone acetylation is broadly associated with transcriptional activation and plays pivotal roles in differentiation [8], development [9], and tumorigenesis [10]. Therefore, clarifying how histone acetylation is established and maintained may provide new strategies for precise epigenetic therapy in cancer.

Histone acetylation is dynamic and reversible, regulated by opposing enzyme classes: acetyltransferases (writers) and deacetylases (erasers) [11]. Histone acetyltransferases transfer acetyl groups from acetyl-CoA to the ε-amino group of lysine residues on histone tails [12], thereby neutralizing lysine’s positive charge and weakening histone-DNA interactions [13], and promoting an open chromatin state that facilitates transcription factor binding and RNA polymerase II recruitment [14]. Conversely, histone deacetylases remove acetyl groups [15], restoring lysine’s positive charge, compacting chromatin, and generally repressing transcription [16]. Mammalian histone deacetylases fall into two major families: the family of classical Zn^2+^-dependent histone deacetylases (HDACs) [17] and the NAD⁺-dependent sirtuins [18]. While the functions and regulation of the HDAC family [19] have been extensively characterized, sirtuins have emerged as important modulators of metabolism [20], genome maintenance [21], and cancer biology. However, the mechanism by which the sirtuin family confers specificity and context-dependent control over histone deacetylation remains unclear.

Long non-coding RNAs (lncRNAs), typically defined as transcripts longer than 200 nucleotides with limited protein-coding ability [22], have transitioned from being considered transcriptional “noise” to recognized regulators of gene expression [23]. They function through diverse mechanisms—scaffolding macromolecular assemblies [24], guiding chromatin regulators [25], acting as molecular decoys, and modulating RNA/protein stability—to control transcription [25], RNA processing, and chromatin states [26]. Accumulating evidence links dysregulated lncRNAs to cancer [27], including roles in epigenetic remodeling. For example, lncRNA SNHG29 restrains FLT3-ITD acute myeloid leukemia by binding to EP300 and altering histone acetylation [28], and LncRNA-PACERR promotes histone acetylation in pancreatic ductal adenocarcinoma through nuclear interaction with KLF12 and recruitment of EP300 [29]. Despite these advances, a key question remains: how do lncRNAs precisely regulate histone acetylation by controlling the activity, localization, or stability of histone acetyltransferases or deacetylases, and thereby dictate cell fate decisions? Addressing this question will be essential for decoding “epigenetic circuitry” and for developing more specific drugs.

We previously identified a long non-coding RNA (Linc00637), named HIF-1α inhibitor at translation level (HITT) [30, 31], whose decreased expression correlated with poor prognosis in colorectal cancer. HITT is a multifunctional lncRNA implicated in distinct intrinsic pathways, including immune regulation (by cooperating with RGS2 to inhibit PD-L1 translation) [32], autophagy (ATG5-dependent) [33], and mitochondrial dynamics (MFN2-dependent) [34]. However, it still remains unknown whether HITT participates in the epigenetic regulation process. Here, guided by bioinformatics analysis linking HITT to histone post-translational modifications, we uncover an epigenetic role for HITT in colorectal cancer cells. We show that HITT directly binds histone deacetylase SIRT6 and stabilizes SIRT6 by disrupting formation of the MDM2-SIRT6 complex, thereby limiting MDM2-dependent ubiquitin-mediated proteasomal turnover of SIRT6. Through this mechanism, HITT selectively suppresses histone H3/H4 acetylation and colorectal cancer cell growth, defining a HITT-SIRT6 regulatory axis that connects lncRNA-dependent protein stabilization to histone acetylation homeostasis.

## Results

### HITT inhibits histone H3/H4 acetylation in a SIRT6-dependent manner

**Fig. 1:**
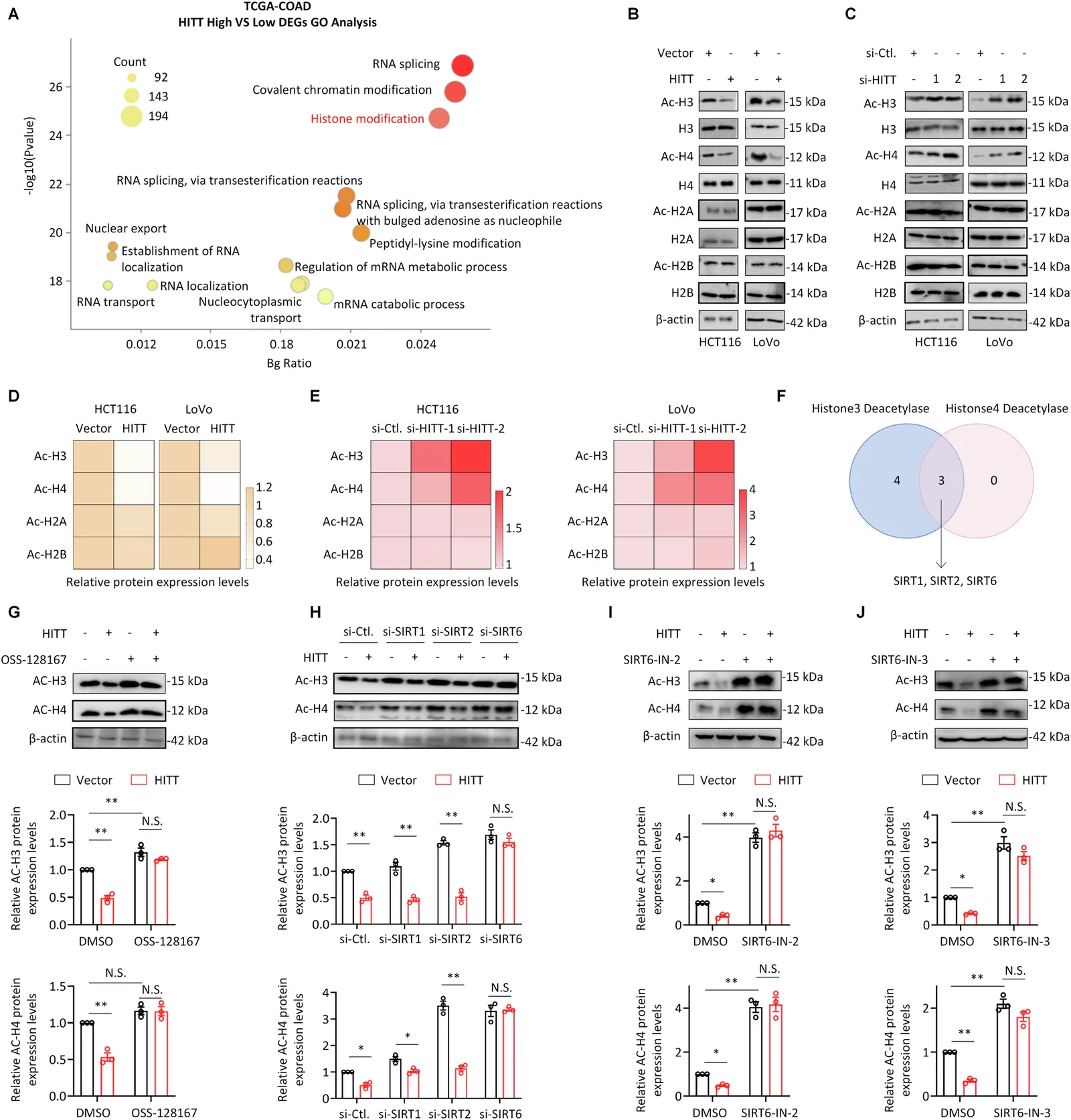
LncRNA HITT inhibits histone H3/H4 acetylation. **A** Gene Ontology (GO) enrichment analysis of differentially expressed genes (DEGs) between the HITT-high and HITT-low groups in the TCGA-COAD dataset. Representative Western blot images showing the protein expression levels and acetylation status of histones H2A, H2B, H3, and H4 in HITT overexpression (OE) (**B**) or HITT knockdown (KD) (**C**) cells. Quantification of the Western blot data for HITT OE is presented in (**D**), while the corresponding quantification for HITT KD is shown in (**E**). **F** Venn diagram illustrating the overlap between H3 deacetylases and H4 deacetylases. **G** Representative Western blot images showing the acetylation levels of histones H3/H4 in HITT OE cells with or without treatment with OSS-128167 (1.5 mM, 24 h) (Top). The images were quantified by ImageJ, and quantification is shown in the bar graphs (lower panel). (**H**) Representative Western blot images showing the acetylation levels of histones H3/H4 in HITT OE cells under conditions of knockdown of SIRT1, SIRT2, or SIRT6 (Top). The images were quantified by ImageJ, and the data were plotted in the bar graphs (Bottom). Representative Western blot images showing the acetylation levels of histones H3/H4 in HITT OE cells, with or without treatment with SIRT6-IN-2 (40 μM, 24 h) (**I**, Top) or SIRT6-IN-3 (10 μM, 24 h) (**J**, Top). The images were quantified using ImageJ, and the quantified data are presented in the bar graphs (**I**, **J**, Bottom). Data are derived from three independent experiments and represented as mean ± SEM in the bar graph. *P < 0.05, **P < 0.01, NS not significant (**G**–**J**).

We previously identified the long non-coding RNA, Linc00637, which we named HITT [30]. Previous studies have shown that HITT regulates multiple intrinsic cellular processes, including immunity, DNA damage responses, and autophagy [33]. To explore the potential epigenetic function of HITT in colorectal cancer, we analyzed the TCGA-COAD cohort by dividing tumors into HITT-high and HITT-low groups and performed Gene Ontology enrichment analysis on the differentially expressed genes. Notably, biological process terms associated with histone modification were significantly enriched in relation to HITT expression (Fig. 1A), suggesting a previously unappreciated link between HITT and chromatin regulation. To verify which histone modifications were affected by HITT expression, we overexpressed (OE) and knocked down (KD) HITT in two colorectal cancer cell lines, HCT116 and LoVo, and detected the canonical modifications on core histones. HITT overexpression did not alter global histone methylation and lactylation (Fig. S1A, B). In contrast, HITT-OE markedly reduced acetylation of histone H3/H4 (Fig. 1B-E), whereas HITT-KD increased histone H3/H4 acetylation (Fig. 1B–E). Acetylation levels of histone H2A and H2B were not affected by manipulating HITT expression (Fig. 1B–E). Together, these results indicate that HITT selectively suppresses histone H3/H4 acetylation.

Histone acetylation is classically governed by the opposing actions of histone acetyltransferases and deacetylases [35]. To explore how HITT modulates H3/H4 acetylation, we curated candidate enzymes reported to regulate H3/H4 acetylation and intersected these lists (Venn plot). Among the deacetylases examined, the sirtuins SIRT1, SIRT2, and SIRT6 emerged as candidates (Fig. 1F), whereas among the acetyltransferases examined, P300, CBP, GCN5L2, and TIP60 were identified as the major candidates (Fig. S2A). We first tested whether HITT acts through acetyltransferases by knocking down each of the four candidates in HCT116 cells followed by HITT overexpression. The results indicate that knockdown of histone acetyltransferases did not alter the ability of HITT-OE to reduce H3/H4 acetylation levels (Fig. S2B). We next examined the involvement of histone deacetylases. Treatment with OSS-128167, an inhibitor targeting SIRT1/2/6, largely rescued the reduction in H3/H4 acetylation induced by HITT overexpression (Fig. 1G), supporting a requirement for sirtuin deacetylase activity.

To pinpoint the responsible sirtuin, we performed siRNA-mediated knockdown of SIRT1, SIRT2, or SIRT6 in HCT116 cells in the context of HITT overexpression. Notably, depletion of SIRT6, but not SIRT1 or SIRT2, abrogated the ability of HITT-OE to reduce H3/H4 acetylation (Fig. 1H), identifying SIRT6 as the critical mediator. Consistently, two independent SIRT6-specific inhibitors yielded similar results (Fig. 1I, J). These results collectively demonstrate that HITT inhibits histone H3/H4 acetylation in a SIRT6-dependent manner.

### HITT directly binds to SIRT6 and stabilizes SIRT6 by restraining ubiquitin-proteasome turnover

**Fig. 2:**
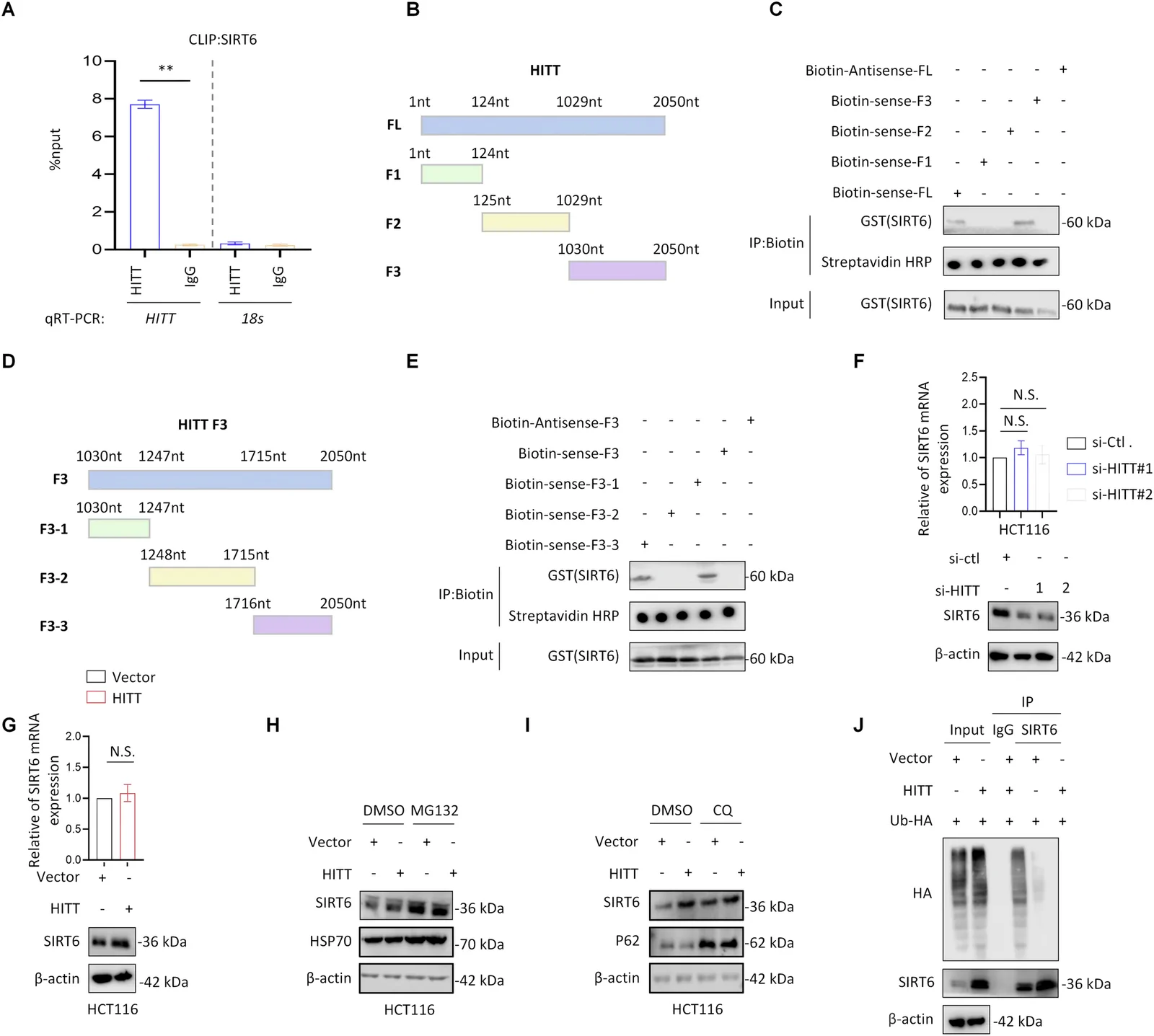
HITT directly binds to SIRT6 and stabilizes SIRT6 by restraining ubiquitin-proteasome turnover. **A** SIRT6-associated HITT was detected by qRT-PCR following CLIP of SIRT6. **B** Truncation of HITT and its labeling. **C** The interaction between biotinylated HITT truncates and GST-SIRT6 was determined by biotinylated RNA pull-down assay. **D** Truncation of HITT-F3 and its labeling. **E** The interaction between SIRT6 and biotinylated HITT-F3 subfragments (F3-1, F3-2, and F3-3) was determined by RNA pull-down assay. Representative Western blot images showing the protein levels of SIRT6 in HITT OE cells (**F**, Bottom) and HITT KD cells (**G**, Bottom). The images were quantified using ImageJ, and the data are presented in the bar graphs (**F**, **G**, Top). SIRT6 protein levels were determined by Western blot in HITT OE cells treated with or without the proteasome inhibitor MG-132 (**H**) (20 μM, 24 h) and the lysosomal inhibitor CQ (**I**) (50 μM, 24 h). **J** Ub-SIRT6 was detected using IP/Western blot assay in HCT116 cells with HITT OE. Data are shown as mean ± SEM (*n* = 3). **P < 0.01, NS not significant (**A**, **F**, **G**).

To elucidate how HITT suppresses histone H3/H4 acetylation through SIRT6, we first asked whether HITT physically associates with SIRT6. The CLIP assay revealed robust enrichment of HITT in SIRT6 immunoprecipitates (Fig. 2A). To map the interaction region, we generated three HITT truncation mutants, designated F1 (1–124 nt), F2 (125–1029 nt), and F3 (1030–2050 nt) (Fig. 2B), and performed in vitro RNA pull-down assays. Full-length HITT and the F3 segment, but not F1 or F2, bound SIRT6 (Fig. 2C). We next subdivided the F3 into three small fragments: F3-1 (1030–1247 nt), F3-2 (1248–1715 nt), and F3-3 (1716–2050 nt) (Fig. 2D). The results indicated that SIRT6 preferentially bound the F3-3 fragment (Fig. 2E). These data identify the 1716–2050 nt region of HITT as the minimal region sufficient for direct binding to SIRT6.

We next examined whether HITT binding influences SIRT6 expression. In HCT116 cells, siRNA-mediated depletion of HITT using two independent siRNAs reduced SIRT6 protein levels (Fig. 2F), whereas HITT-OE increased SIRT6 protein abundance (Fig. 2G). Notably, SIRT6 mRNA levels did not change on either HITT knockdown or overexpression (Fig. 2F, G), suggesting a post-transcriptional mechanism. To define the pathway responsible for SIRT6 regulation, we overexpressed HITT in HCT116 cells and treated cells with the proteasome inhibitor MG-132 or the lysosomal inhibitor chloroquine (CQ). MG-132 largely abolished the HITT-induced increase in SIRT6 protein (Fig. 2H), while CQ had little effect (Fig. 2I), implicating the ubiquitin-proteasome system rather than lysosomal degradation. Consistently, ubiquitination assays showed that HITT reduced ubiquitination of SIRT6 (Fig. 2J). Overall, these results demonstrate that HITT directly binds to SIRT6 and enhances SIRT6 protein stability by limiting ubiquitin-dependent proteasomal degradation.

### HITT antagonizes MDM2-dependent ubiquitination and degradation of SIRT6

Given the stabilizing effect of HITT on SIRT6, we further analyzed the clinical relevance of SIRT6 in colorectal cancer. Consistent with reduced HITT expression, SIRT6 was also significantly down-regulated in colorectal cancer samples (Fig. S3A) and higher SIRT6 expression positively correlated with favorable patient prognosis (Fig. S3B), supporting a potential tumor-suppressive role.

**Fig. 3:**
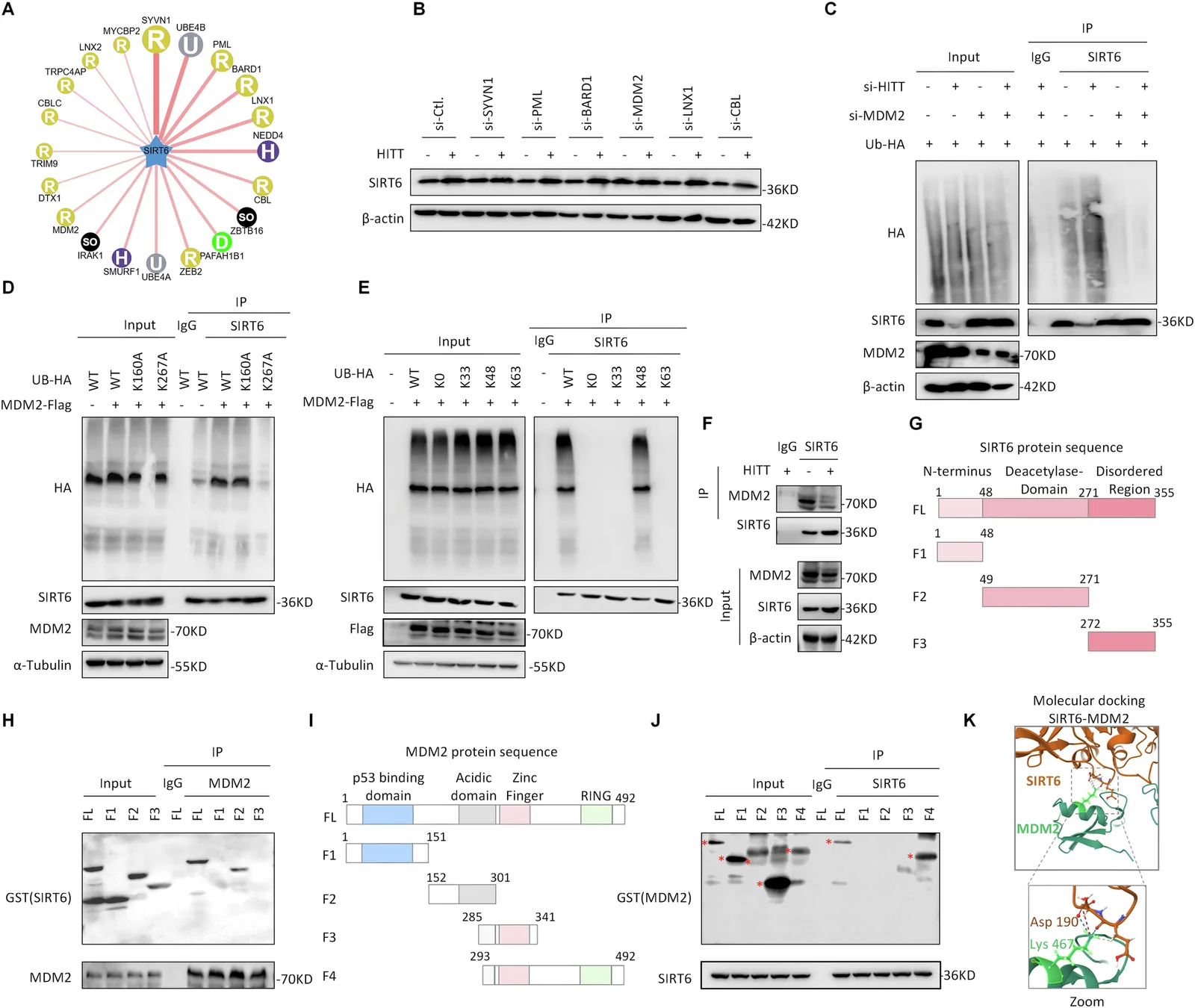
HITT antagonizes MDM2-dependent ubiquitination and degradation of SIRT6. **A** Potential E3 ubiquitin ligases for SIRT6 predicted by the UbiBrowser database. **B** The protein levels of SIRT6 were determined by Western blot after KD of E3 ligase candidates. **C** Ub-SIRT6 was determined by IP/Western blot in HCT116 cells in the presence or absence of HITT KD and/or MDM2 KD. **D** Ub-WT-SIRT6 or Ub-SIRT6 mutants at the predicted ubiquitination sites were determined by the IP/Western blot assay in MDM2-Flag OE HCT116 cells. **E** Polyubiquitin chains with K0, K33, K48, or K63 linkage of SIRT6 were determined by IP/Western blot assay in HCT116 cells transfected with MDM2-Flag. **F** IP assays were performed to compare the binding between MDM2 and SIRT6 in cells with or without HITT OE. **G** Truncation of SIRT6 and its labeling. **H** The interaction between full-length (FL) MDM2 and SIRT6 truncation mutants (F1, F2, and F3) was evaluated by an in vitro binding assay. **I** Truncation of MDM2 and its labeling. **J** The interaction between full-length (FL) SIRT6 and MDM2 truncation mutants (F1, F2, F3, and F4) was evaluated by an in vitro binding assay. **K** A predicted structural model showing the interaction between SIRT6 (orange) and MDM2 (green).

Subsequently, we sought to identify the E3 ubiquitin ligase responsible for SIRT6 ubiquitination and determine whether it mediates the HITT effect. Candidate E3 ligases were first predicted using the UbiBrowser online database (Fig. 3A). E3 ubiquitin ligases, which confer substrate specificity during ubiquitination, are broadly classified into two major families based on their catalytic domains: RING (Really Interesting New Gene)-domain E3 ligases, which function as scaffolds that directly transfer ubiquitin from an E2-conjugating enzyme to the substrate, and HECT (Homologous to E6-AP Carboxyl Terminus)-domain E3 ligases, which first accept ubiquitin onto a catalytic cysteine residue before transferring it to the substrate [36]. Pharmacological inhibition further supported involvement of a RING-type E3 ligase. MLN4924, an inhibitor of the NEDD8-activating enzyme that selectively blocks the activity of Cullin-RING-type E3 ligases, largely abolished the increase in SIRT6 protein induced by HITT overexpression, whereas Heclin, a HECT-domain E3 ligase inhibitor, had no such effect (Fig. S3C). We therefore knocked down the top-ranked RING-type E3 ubiquitin ligases from Fig. 3A along with MDM2, a previously reported E3 ligase for SIRT6 [37]. Notably, depletion of MDM2 eliminated the effect of HITT overexpression on SIRT6 protein abundance (Fig. 3B). Consistently, ubiquitination assays in HCT116 cells further indicated that MDM2 is a key E3 ubiquitin ligase through which HITT restrains SIRT6 ubiquitination (Fig. 3C). To map the ubiquitination site(s) on SIRT6, we used the UbiBrowser database and found two candidate lysines: K160 and K267 (Fig. S3D), and generated lysine to alanine mutants. Ubiquitination assays indicated that mutation of K267 markedly reduced SIRT6 ubiquitination (Fig. 3D), identifying K267 as a major ubiquitination site. Moreover, SIRT6 polyubiquitination was predominantly K48-linked (Fig. 3E), consistent with proteasomal targeting.

Importantly, as a binding partner of SIRT6, HITT-OE significantly weakened the binding of SIRT6 to MDM2 (Fig. 3F), suggesting that HITT stabilizes SIRT6 by disrupting the formation of the MDM2-SIRT6 complex. We generated SIRT6 truncation mutants corresponding to three distinct regions—F1 (N-terminus, amino acids 1-48), F2 (deacetylase domain, amino acids 49-271), and F3 (C-terminal disordered region, amino acids 272-355) (Fig. 3G)—and performed co-immunoprecipitation (Co-IP) assays. The F2 deacetylase domain of SIRT6 directly bound to the full-length (FL) MDM2 (Fig. 3H). Similarly, domain mapping of MDM2 using truncations (F1, 1-151; F2, 152-301; F3, 285-341; F4, 293-492) (Fig. 3I) revealed that the F4 region of MDM2 bound to purified full-length SIRT6 (Fig. 3J). Structural modeling based on AlphaFold v3 and docking simulation further highlighted putative key contact residues—Asp190 in the F2 region of SIRT6 and Lys467 in the F4 region of MDM2 (Fig. 3K). We next asked which region of SIRT6 is responsible for HITT binding. CLIP assays using Flag-tagged full-length SIRT6 and the same truncation mutants revealed that HITT was robustly enriched in immunoprecipitates of full-length SIRT6 and the F2 deacetylase domain, but not in those of the N-terminal F1 or C-terminal F3 regions (Fig. S3F), indicating that HITT and MDM2 engage the same domain of SIRT6. Consistent with this, in vitro RNA pull-down assays indicated that HITT had no direct binding to MDM2 (Fig. S3E). To directly test whether HITT competes with MDM2 for binding to SIRT6 in a cellular context, we co-transfected HCT116 cells with a fixed amount of Flag-MDM2 and increasing doses of an HITT-overexpression plasmid, followed by anti-Flag immunoprecipitation and detection of co-precipitated endogenous SIRT6. The amount of SIRT6 co-immunoprecipitated with Flag-MDM2 progressively decreased with increasing HITT expression, whereas an unrelated control lncRNA had no such effect (Fig. S3G), demonstrating that HITT dose-dependently displaces MDM2 from SIRT6. Together, these data support a model in which HITT directly engages the deacetylase domain of SIRT6 and thereby limits MDM2 access to SIRT6, reducing ubiquitin-dependent degradation.

### SIRT6 agonist MDL-800 induces HITT expression by upregulation of transcription factor E2F1

**Fig. 4:**
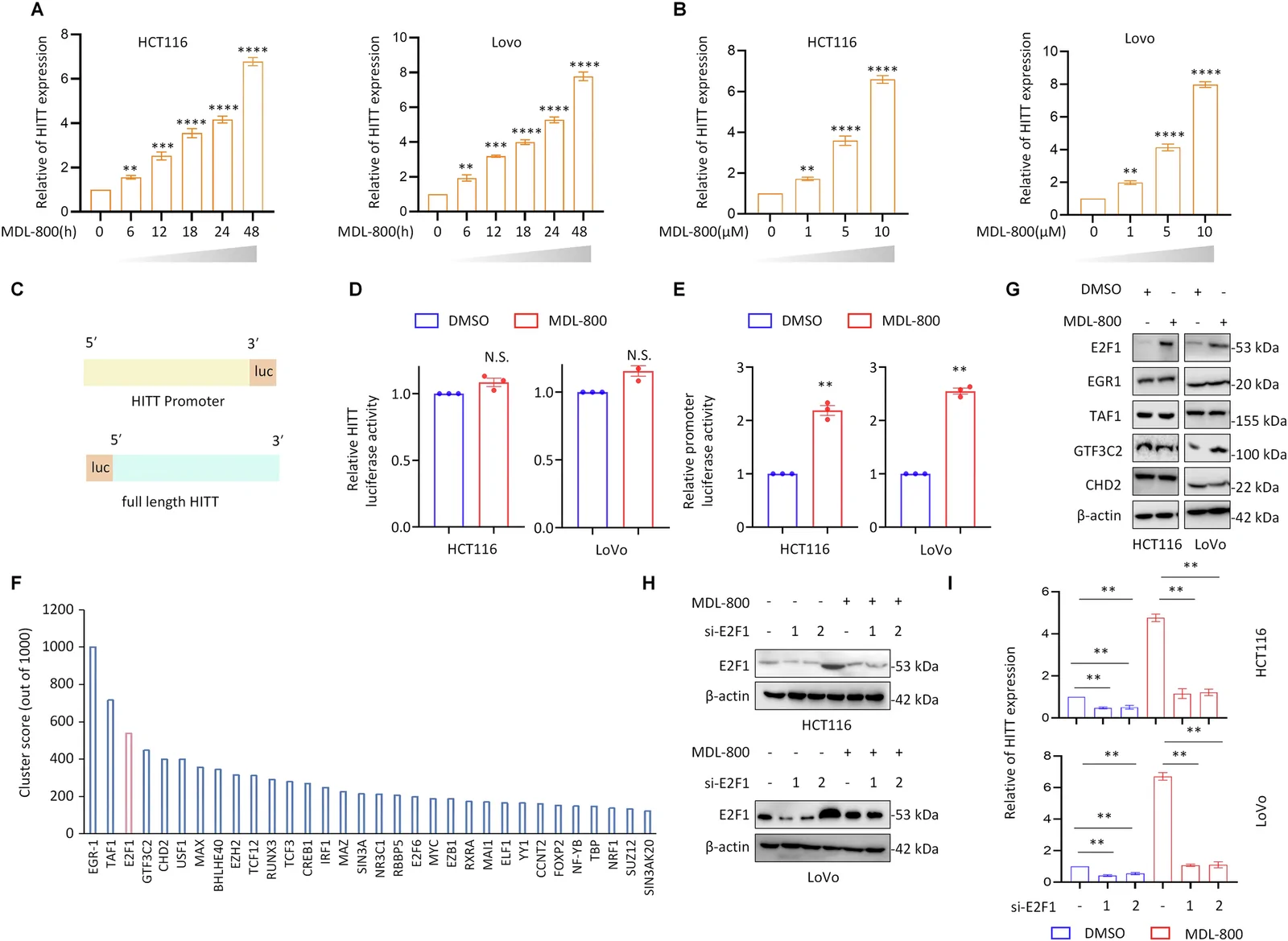
SIRT6 agonist MDL-800 induces HITT expression by upregulation of transcription factor E2F1. **A** qRT-PCR analysis of HITT expression in HCT116 (left) and LoVo (right) cells treated with MDL-800 (0.1 mM) for the indicated time durations. **B** qRT-PCR analysis of HITT expression in HCT116 (left) and LoVo (right) cells treated with different concentrations of MDL-800. **C** Schematic diagram of HITT and the HITT promoter–driven luciferase reporter. (D, E) Luciferase reporter assays measuring HITT 3’-UTR-dependent regulation (**D**) and HITT promoter activity (**E**) in HCT116 and LoVo cells treated with or without MDL-800 (0.1 mM, 24 h). **F** Relative transcription factor binding potentials at the HITT promoter were analyzed using UCSC ChIP-seq data. **G** Western blot analysis of E2F1, EGR1, TAF1, GTF3C2, and CHD2 protein levels in HCT116 and LoVo cells treated with DMSO or MDL-800 (0.1 mM, 24 h). β-actin served as the loading control. **H** Western blot of E2F1 protein levels in HCT116 (top) and LoVo (bottom) cells transfected with control siRNA (–) or two independent siRNAs against E2F1 (si-E2F1#1 or si-E2F1#2), followed by treatment with vehicle (–) or MDL-800 (0.1 mM, 24 h). β-actin served as the loading control. **I** qRT-PCR analysis of HITT expression in HCT116 (top) and LoVo (bottom) cells transfected with control siRNA (–) or two independent siRNAs against E2F1 (si-E2F1#1 or si-E2F1#2), followed by treatment with DMSO (vehicle) or MDL-800 (0.1 mM, 24 h). Data are shown as mean ± SEM (*n* = 3). **P < 0.01, NS not significant (**A**, **B**, **E**, **I**).

HITT is downregulated in colorectal cancer [30], prompting us to investigate upstream regulators that control HITT expression in the context of histone-modification pathways. Given our finding that HITT functionally engages SIRT6, we asked whether pharmacologic activation of SIRT6 influences HITT expression. Treatment of HCT116 and LoVo cells with the SIRT6 agonist, MDL-800 increased HITT levels in both a time- and dose-dependent manner (Fig. 4A, B).

To distinguish between transcriptional and post-transcriptional regulation, we constructed two luciferase reporters to assess HITT promoter activity and 3’-untranslated region (3’-UTR)-dependent regulation, respectively (Fig. 4C). MDL-800 had minimal effect on HITT 3’-UTR reporter (Fig. 4D), whereas it markedly increased luciferase activity driven by the HITT promoter (Fig. 4E). These data indicate that MDL-800 induces HITT primarily at the transcriptional level.

Next, we sought to identify transcription factors mediating this induction. Candidate transcriptional regulators were predicted using the UCSC database (Fig. 4F). Among the top-ranked candidates, MDL-800 treatment selectively increased E2F1 protein expression, whereas the other candidates showed little or no change (Fig. 4G). Consistently, E2F1 levels gradually increased in a time- and dose-dependent manner upon MDL-800 exposure (Fig. S4A, B), suggesting that E2F1 might link SIRT6 activation to HITT transcription. To test this, we depleted E2F1 using two independent siRNAs in HCT116 and LoVo cells (Fig. 4H). Notably, E2F1 knockdown completely abrogated the MDL-800-mediated induction of HITT (Fig. 4I). These data demonstrate that MDL-800 activates HITT transcription through upregulation of the transcription factor E2F1.

### Targeting HITT-SIRT6 axis suppresses colorectal cancer growth in vitro and in vivo

**Fig. 5:**
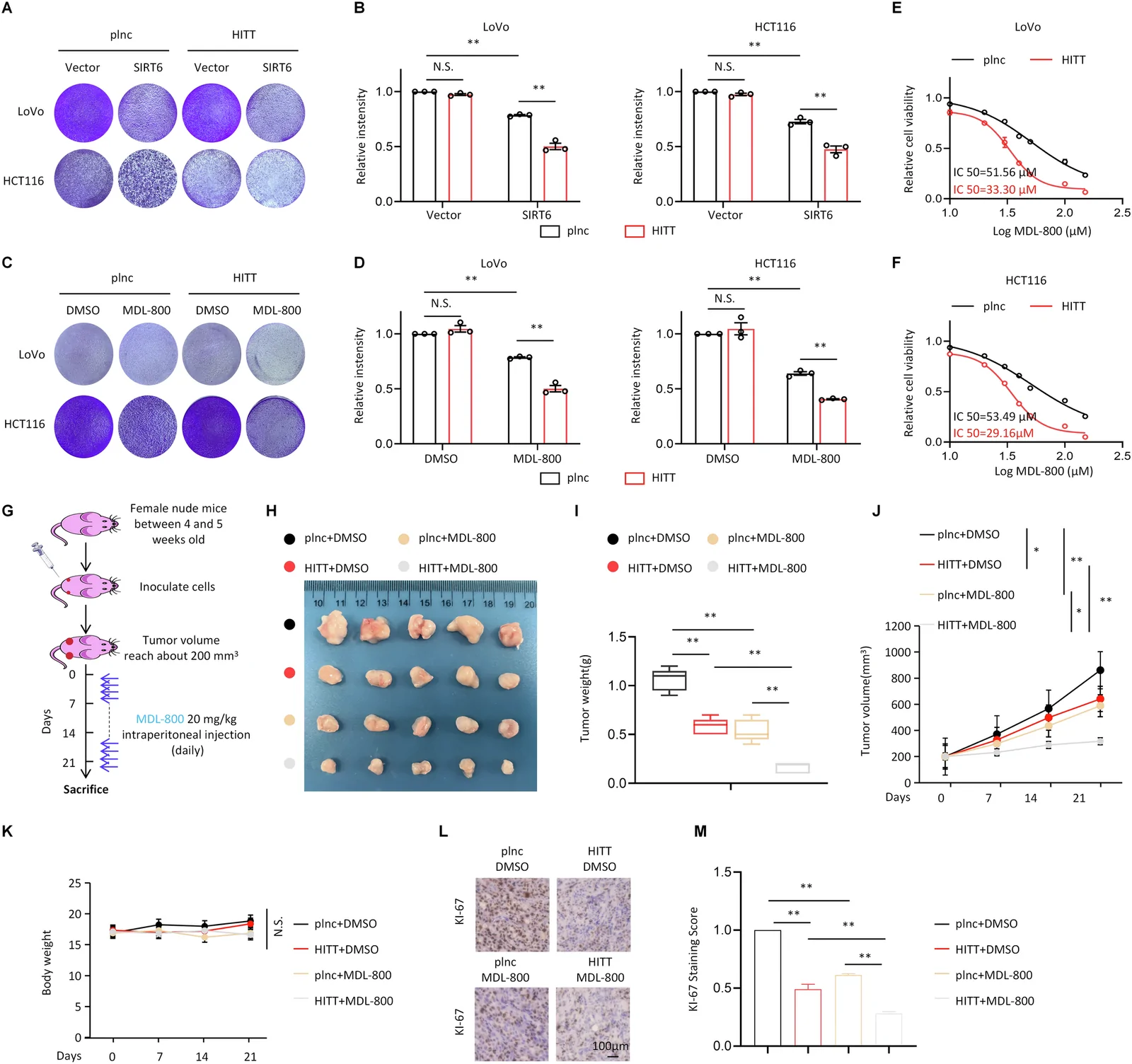
Targeting the HITT-SIRT6 axis suppresses colorectal cancer growth in vitro and in vivo. **A**–**D** Crystal violet staining of HCT116 and LoVo cells with the indicated treatments. Representative images (**A**, **C**) and quantification (**B**, **D**) are shown. **E**, **F** Cell viability curves (CCK-8) showing the IC50 of MDL-800 (0.1 mM, 24 h) in empty vector control (plnc) and HITT OE CRC cells. **G** Experimental schematic for cell inoculation and MDL-800 treatment. Representative images of subcutaneous tumors (**H**), tumor weights (**I**), and tumor volume growth curves (**J**). **K** Changes in mouse body weights during treatment. Representative images (**L**) and statistical analysis (**M**) of Ki-67 immunohistochemical staining in tumor tissues. Scale bar = 100 μm. Data represent mean ± SEM (**A**–**F**); Data represent mean ± SD (**H**–**M**); *P < 0.05, **P < 0.01, NS not significant (**B**, **D**, **I**, **J**, **K**, **M**).

Histone acetylation is a key epigenetic determinant of cancer cell proliferation and survival [38]. Therefore, we assessed whether manipulating the HITT-SIRT6 axis influences colorectal cancer cell growth. HITT and/or SIRT6 were overexpressed in HCT116 and LoVo cells, and cell proliferation was initially evaluated by crystal violet staining, followed by CCK-8 dose–response assays to assess MDL-800 sensitivity. The results showed that HITT-OE alone had minimal effect on cell growth, whereas SIRT6-OE alone markedly suppressed proliferation. Of note, co-expression of HITT and SIRT6 further enhanced growth inhibition compared with SIRT6 overexpression alone (Fig. 5A, B). Consistent with these findings, combining HITT-OE with the SIRT6 agonist MDL-800 produced a stronger anti-proliferative effect than either intervention alone (Fig. 5C, D). In addition, CCK-8 assays indicated that HITT-OE increased cellular sensitivity to MDL-800 by ~1.5-fold in both colorectal cancer cells (Fig. 5E, F).

To evaluate the therapeutic relevance of this axis in vivo, we established a cell line-derived xenograft (CDX) model using HCT116 cells stably overexpressing HITT, as previously reported [30]. Control and HITT-OE HCT116 cells were implanted subcutaneously into opposite flanks of BALB/c nude mice. When tumors reached comparable volumes (approximately 200 mm^3^), mice were randomized to the indicated treatments (*n* = 5 per group) (Fig. 5G). Either HITT overexpression or MDL-800 treatment alone significantly reduced tumor volume and tumor weight (Fig. 5H–J). Strikingly, combined HITT-OE and MDL-800 treatment produced a substantially greater reduction in tumor burden, yielding an approximately 4-5 fold decrease relative to untreated controls (Fig. 5H–J). MDL-800 was well tolerated, as reflected by unchanged body weight (Fig. 5K). Immunohistochemical staining further indicated that Ki-67 positively tracked with tumor size (Fig. 5L, M). Collectively, these data indicate that activating the HITT-SIRT6 axis sensitizes colorectal cancer cells to MDL-800 and suppresses tumor growth, both in vivo and in vitro.

To further validate the proposed lncRNA-proteostasis-chromatin axis in vivo, we examined SIRT6 protein levels and histone H3/H4 acetylation status in tumor tissues. Western blot analyses of protein lysates from xenograft tumors confirmed that SIRT6 protein was elevated in both HITT-OE and MDL-800-treated tumors relative to the vector control, with the most pronounced increase observed in the HITT-OE + MDL-800 combination group (Fig. S5A). Consistent with enhanced SIRT6 stabilization, acetylation of histone H3 and H4 was progressively reduced across the single-treatment groups and was nearly abolished in the combination group (Fig. S5A). qRT-PCR analysis of the same tumor tissues further confirmed efficient HITT overexpression in vivo, and demonstrated that MDL-800 treatment significantly induced HITT expression, with the combination group exhibiting the highest HITT levels (Fig. S5B). Together, these in vivo findings are fully consistent with the in vitro mechanism and validate the functional integrity of the HITT-SIRT6-H3/H4 acetylation axis.

## Discussion

**Fig. 6:**
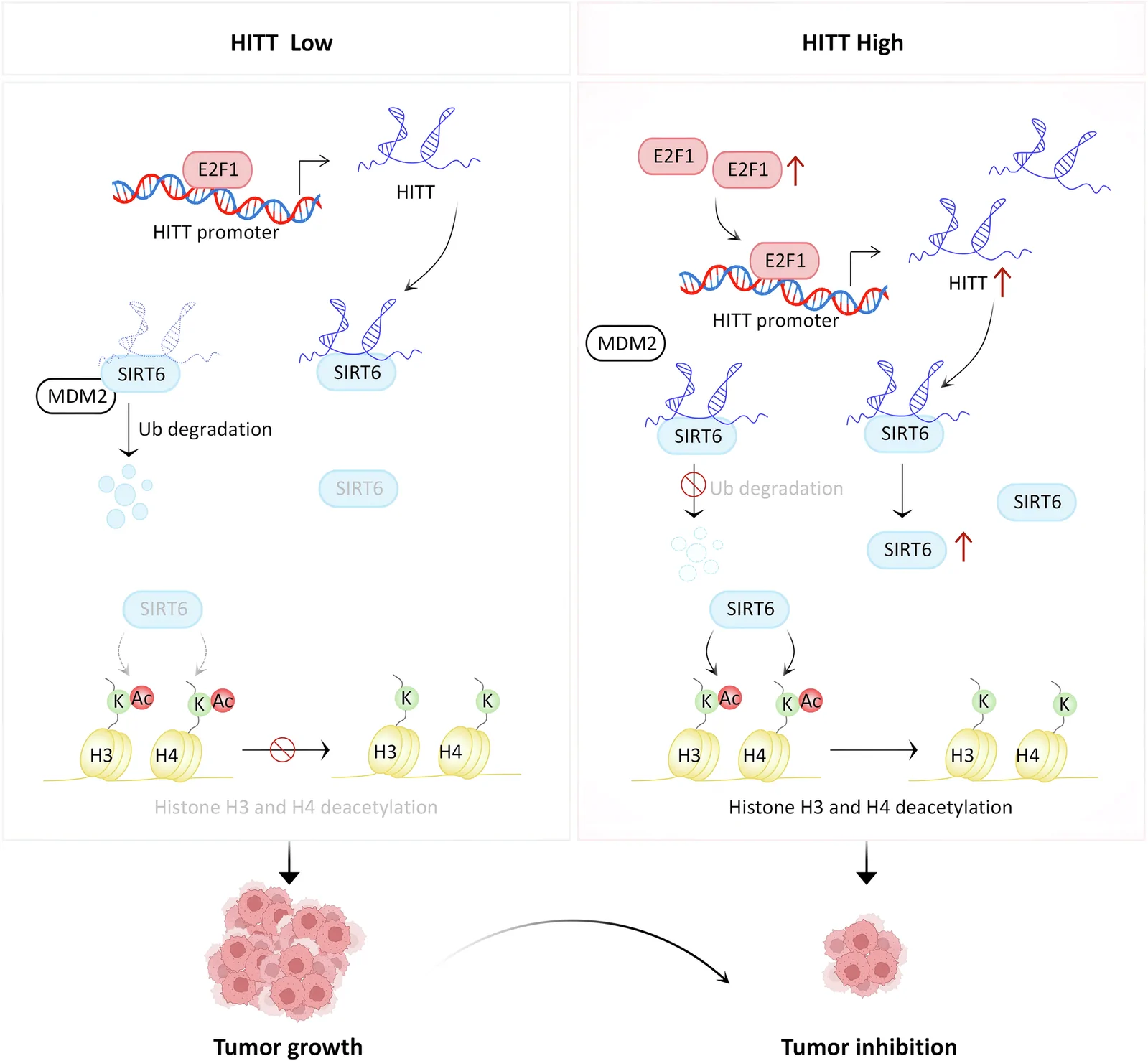
Proposed model: LncRNA HITT inhibits histone H3/H4 acetylation by stabilizing the deacetylase SIRT6. Under basal HITT expression, E2F1 occupies the HITT promoter and sustains its constitutive transcription. Although HITT is capable of interacting with SIRT6, SIRT6 is preferentially targeted for MDM2-dependent ubiquitination and proteasomal degradation, resulting in diminished SIRT6 protein abundance. Reduced SIRT6 levels compromise its histone deacetylase activity toward histones H3/H4, leading to elevated histone acetylation and thereby facilitating tumor progression. Conversely, upon robust E2F1-driven transcriptional activation of HITT, HITT expression is markedly increased. Abundant HITT directly associates with SIRT6 and interferes with its interaction with MDM2, effectively blocking SIRT6 ubiquitination and subsequent degradation. This stabilization and accumulation of SIRT6 enhances H3/H4 deacetylation, ultimately suppressing tumor cell growth.

In this study, we uncover a previously unappreciated epigenetic function of the long non-coding RNA HITT in colorectal cancer. Our data support a model in which HITT serves as a stability regulator for the sirtuin deacetylase SIRT6, thereby constraining histone H3/H4 acetylation and determining cancer cell fate. Mechanistically, pharmacologic activation of SIRT6 by MDL-800 increases the abundance of the transcription factor E2F1, which in turn induces HITT transcription. HITT then physically binds to SIRT6 through its 1716-2050 nt region and limits formation of the MDM2-SIRT6 complex, reducing MDM2-dependent K48-linked ubiquitination and proteasomal turnover of SIRT6. Stabilized SIRT6 is required for the HITT-dependent suppression of histone H3/H4 acetylation, and activation of this axis sensitizes colorectal cancer cells to MDL-800, resulting in enhanced growth inhibition in vitro and reduced tumor burden in vivo (Fig. 6). Together, these findings establish HITT as a lncRNA-encoded stability switch that links ubiquitin control of SIRT6 to histone acetylation homeostasis and tumor suppression.

Histone acetylation is a central determinant of chromatin accessibility and transcriptional output [39], and dysregulated acetylation is widely implicated in oncogenic programs [40]. Our results identify the HITT-SIRT6 axis as an important regulator of H3/H4 acetylation, with an intriguing degree of selectivity: altering HITT levels robustly affects the acetylation of H3/H4, while leaving H2A and H2B acetylation largely unchanged. This selectivity may reflect substrate preference and chromatin engagement modes of SIRT6, but it also raises an important question regarding downstream transcriptional consequences. Although we establish a clear link between HITT, SIRT6 stability, and global H3/H4 acetylation, the precise genomic loci and gene networks controlled by this pathway remain to be mapped. Future research combining ChIP-seq for key acetylation sites (e.g., H3K9ac, H3K27ac, and H4K16ac) with transcriptomic profiling (RNA-seq) will be valuable to delineate the direct epigenetic targets and identify the transcriptional programs that mediate growth suppression upon HITT-SIRT6 activation.

A broader conceptual advance from this work is the mechanism by which HITT regulates chromatin. LncRNAs are often viewed as static “recruitment scaffolds” that recruit chromatin regulators to defined genomic loci [41]. For instance, lncRNA HOTAIR regulates gene expression by recruiting PRC2 complexes to specific genomic loci [42]. Our data extend this framework by showing that HITT regulates chromatin not by recruiting a chromatin modifier to specific genomic loci, but by stabilizing the deacetylase SIRT6 through competitive disruption of MDM2-dependent ubiquitination. This “stability-switch” mode of regulation expands the functional repertoire of lncRNAs beyond locus-specific recruitment and highlights protein quality control as an underappreciated entry point for lncRNA-mediated epigenetic remodeling. Our data support a competitive binding model in which HITT and MDM2 engage the same deacetylase domain (F2 region) of SIRT6. Dose-dependent HITT overexpression progressively displaces MDM2 from SIRT6 in cells (Fig. S3G), while HITT itself does not directly bind MDM2 (Fig. S3E). Together, these results indicate that HITT stabilizes SIRT6 primarily by competing with MDM2 for access to the deacetylase domain, thereby protecting SIRT6 from MDM2-mediated ubiquitination and proteasomal degradation. While our current data strongly favor a competitive mechanism, high-resolution structural studies will be needed to determine whether the HITT and MDM2 binding surfaces on SIRT6 are fully overlapping or partially distinct.

Targeting epigenetic modifiers, particularly histone deacetylases, has emerged as a promising anti-cancer strategy [43]. However, broad-spectrum HDAC inhibitors [44] have shown anti-cancer activity but are frequently limited by toxicity and imperfect specificity. Our findings suggest an alternative strategy: enhancing the function of a defined tumor-suppressive deacetylase, rather than globally inhibiting deacetylation. SIRT6 plays a significant role in genomic integrity, metabolism, and cellular senescence. Notably, SIRT6 expression is significantly downregulated in colorectal cancer tissues, yet higher expression levels are associated with favorable patient prognosis, consistent with a tumor-suppressive role. Importantly, our data indicate that activating HITT cooperates with the SIRT6 agonist MDL-800 to produce stronger anti-tumor effects than either intervention alone. This synergy is mechanistically coherent: MDL-800 induces HITT transcription upstream (via E2F1), while HITT overexpression stabilizes SIRT6 protein downstream by limiting MDM2-mediated degradation, thereby amplifying SIRT6-dependent chromatin effects. Such axis-reinforcing combination logic may enable more targeted epigenetic therapy with improved therapeutic windows.

Several questions remain. First, while our results support an E2F1-dependent transcriptional mechanism for MDL-800-induced HITT upregulation, defining direct promoter occupancy and the relevant cis-regulatory elements will strengthen this model. Second, although we establish K267 as a major ubiquitin acceptor site and identify K48-linked ubiquitination as the dominant chain type, the full landscape of SIRT6 ubiquitination and potential deubiquitinases that counterbalance MDM2 remains to be explored. Third, as our in vivo evidence is based on a CDX model, future studies in more clinically relevant systems, such as patient-derived organoids or xenografts, will be important to evaluate therapeutic efficacy and heterogeneity. Finally, SIRT6 is known to act on non-histone substrates, and it will be of interest to determine whether HITT also influences non-histone deacetylation programs that contribute to tumor suppression.

Beyond colorectal cancer, the HITT-SIRT6 axis may represent a more general regulatory principle. SIRT6 dysregulation has been reported across multiple tumor types, and available datasets suggest that HITT expression is perturbed in several cancers. It will therefore be important to test whether HITT similarly stabilizes SIRT6 in other contexts and whether this axis exerts conserved tumor-suppressive functions. If so, strategies aimed at reinforcing the HITT-SIRT6 circuit—through lncRNA activation approaches or small-molecule modulation—could have broader translational potential.

In summary, we define a lncRNA-proteostasis-chromatin axis in which HITT stabilizes SIRT6 by antagonizing MDM2-dependent turnover, thereby constraining H3/H4 acetylation and inhibiting tumor growth. These findings expand our understanding of how lncRNAs interface with proteostasis to regulate chromatin and lay a foundation for future translational development of targeted epigenetic interventions.

## Methods and Details

### Cell culture and transfection

Human colorectal cancer (HCT116, LoVo) cell lines were purchased from the American Type Culture Collection (ATCC). Cells were cultured in RPMI-1640 or DMEM medium supplemented with 10% fetal bovine serum (FBS) and maintained in a humidified incubator at 37 °C with 5% CO_2_. For transient transfection, synthesized small interfering RNA (siRNA) (Genepharma, China) or plasmids were transfected into target cells using Lipofectamine 2000 or a non-liposomal transfection reagent. Each transfection group included a corresponding control group transfected with an empty plasmid or scrambled RNA. Cells were subjected to subsequent assays 48–72 hours post-transfection. The siRNA sequences involved in this study are presented in Supplementary Table S1.

### RNA extraction and reverse transcription quantitative polymerase chain reaction (qRT-PCR)

Cells were washed three times with 1× phosphate-buffered saline (PBS) to remove residual culture medium. Total RNA was extracted using Trizol reagent (Takara). According to the manufacturer’s instructions (PrimeScript™ RT reagent Kit with gDNA Eraser), 2 μg of purified RNA was used for reverse transcription to synthesize cDNA. Subsequently, quantitative PCR was performed using SYBR Premix Ex Taq™ II (RR820L; Takara) on a Thermo Fisher real-time PCR system. 18S rRNA was used as the internal control, and gene expression was analyzed using the 2^−ΔΔCT^ method. The RT-PCR primer sequences involved in this study are shown in Supplementary Table S2.

### Western Blot (WB) analysis

Collected cell or tissue samples were lysed using urea lysis buffer (8 M urea, 1 M thiourea, 0.5% CHAPS, 50 mM DTT, and 24 mM spermine). Samples were fully disrupted using an ultrasonic homogenizer and vortexed for 30 minutes. Proteins were separated by sodium dodecyl sulfate-polyacrylamide gel electrophoresis (SDS-PAGE) and transferred onto polyvinylidene difluoride (PVDF) membranes. Membranes were blocked with 5% non-fat milk for 1 hour, followed by incubation with primary antibodies against target proteins and corresponding secondary antibodies. Protein signals were visualized using enhanced chemiluminescence (ECL) substrate (32106, Thermo Scientific), and images were captured using the Image Studio System (Li-COR, Lincoln, Georgia, USA). The product sources and dilution ratios of all the primary antibodies used in this experiment are shown in Supplementary Table S3.

### Co-immunoprecipitation (Co-IP) Assay

Cells were lysed with NETN lysis buffer (50 mM Tris-HCl pH 8.0, 150 mM NaCl, 1% NP-40, 1 mM EDTA) supplemented with protease inhibitor cocktail (PIC) (MedChemExpress, USA). Lysates were sonicated to ensure complete protein extraction. Protein G agarose beads (GE Healthcare, USA) were used to pre-clear the lysates by rotating at 4 °C for 1 hour. Pre-cleared lysates were then incubated with blocked Protein G agarose beads and antibodies against the target protein or control IgG antibodies overnight at 4 °C with rotation. Immunoprecipitated beads were collected, washed four times with ice-cold NETN buffer, boiled in SDS loading buffer for 5 minutes, and analyzed by Western blot.

### Ultraviolet Crosslinking RNA Immunoprecipitation (CLIP)

Cells were washed three times with 1× PBS to remove residual medium and then crosslinked with 0.4 J/cm² of UV light (254 nm). Crosslinked cells were lysed in lysis buffer (50 mM Tris-HCl [pH 8.1], 85 mM KCl, 10 mM EDTA, 5 mM PIPES [pH 8.0], 1% SDS, 0.5% NP-40) supplemented with protease inhibitor cocktail (PIC) and RNase inhibitor (MedChemExpress, USA). Lysates were pre-cleared with Protein G agarose beads (GE Healthcare, USA) at 4 °C for 1 hour with rotation. The supernatant was then incubated with blocked Protein G agarose beads and antibodies against the target protein or control IgG overnight at 4 °C. The next day, protein-RNA-bead complexes were collected and further incubated with blocked Protein A/G agarose beads for 1 hour. RNA was eluted, reverse-transcribed into cDNA, and analyzed by qRT-PCR.

### In vitro RNA pull-down assay

Biotin-labeled RNA probes were synthesized in vitro using the Biotin RNA Labeling Mix (Roche, St Louis, MO, USA, 11685597910). After DNase I treatment, RNA probes were heated at 70 °C for 2 minutes and then placed on ice for 3 minutes to renature. Purified GST-tagged protein (10pmol) was mixed with 1 μg of biotin-labeled RNA probe and incubated at 4 °C with rotation for 2 hours. Meanwhile, Streptavidin Magnetic Beads (MedChemExpress, USA) were blocked with blocking buffer (2 mg/ml BSA, 1 mg/ml yeast tRNA) for 1 hour. The protein-RNA mixture was then incubated with the blocked Streptavidin Magnetic Beads at 4 °C for 1 hour with rotation. Beads were collected, washed, boiled in SDS loading buffer for 5 minutes, and analyzed by Western blot.

### Recombinant protein purification

Plasmids containing the recombinant protein gene were transformed into ER2566 expression-competent cells and cultured to the logarithmic growth phase. Protein expression was induced by adding 0.1 mM isopropyl β-D-1-thiogalactopyranoside (IPTG), followed by overnight incubation at 16 °C. Bacterial cells were harvested by centrifugation, and the pellet was resuspended in NETN lysis buffer containing protease inhibitors. Cells were lysed by sonication. The lysate was incubated with 30 μL of BeyoGold™ GST-Tag Purification Resin (Beyotime, China) at 4 °C for 4-6 hours with rotation. Beads were washed three times with NETN buffer, and GST-tagged proteins were eluted with reduced glutathione (GSH) elution buffer.

### Luciferase reporter assay

Luciferase reporter plasmids and Renilla-luciferase control plasmid were co-transfected into cells using a non-liposomal transfection reagent. Forty-eight hours post-transfection, cells were lysed with luciferase lysis buffer. Dual-luciferase reporter assays were performed according to the manufacturer’s instructions. Luciferase activity was calculated as the ratio of firefly luciferase activity to Renilla luciferase activity.

### Crystal violet staining

Cells were seeded in 24-well plates and transfected with HITT overexpression plasmid, SIRT6 plasmid, or corresponding control plasmids using a non-liposomal transfection reagent. Twenty-four hours post-transfection, cells were treated with MDL-800. After 48 hours, cells were washed three times with 1× PBS to remove dead floating cells. Remaining adherent cells were fixed with 4% paraformaldehyde for 30 minutes and then stained with 0.1% crystal violet solution for 20 minutes. After washing three times with PBS, plates were photographed and quantified.

### Ubiquitination assay

HCT116 cells were transfected with an HITT overexpression plasmid and a SIRT6 plasmid using a non-liposomal transfection method. Forty-eight hours post-transfection, MG-132 was added to a final concentration of 15 μM for 6 hours prior to harvesting. Cells were washed twice with 1× PBS and lysed with 100 μL of whole-cell lysis buffer, followed by scraping. Samples were heated at 100 °C for 10 minutes in a metal bath. Ultrasonication was performed four times (15 seconds each, 10-second intervals, 35% power). Then, 900 μL of dilution buffer containing protease inhibitors (1:100) was added, and samples were incubated at 4 °C with rotation for 1 hour. After centrifugation at 16,000 *g* for 30 minutes at 4 °C, protein concentration was measured, and 50 μL was saved as the input sample. For immunoprecipitation, 2 mg of protein lysate was mixed with 1.5 μL of antibody and 20 μL of blocked beads and incubated at 4 °C with rotation for at least 20 hours. Beads were washed four times with dilution buffer, centrifuged at 1,000 g for 5 minutes at 4°C each time. Finally, 30 μL of 2× loading buffer was added, and samples were boiled at 100°C for 5 minutes before Western blot analysis.

### Immunohistochemistry

Paraffin-embedded tissue sections from four treatment groups were baked at 60°C for 2 hours. Sections were deparaffinized by sequential immersion in xylene I and xylene II for 10 minutes each, followed by rehydration through a graded ethanol series (100% ethanol I, 100% ethanol II, 95%, 85%, and 75% ethanol, 5 minutes each) to distilled water. Antigen retrieval was performed by high-pressure heating in citrate buffer (pH 6.0) for 15 minutes, followed by cooling to room temperature. Endogenous peroxidase activity was blocked by incubating with 3% hydrogen peroxide for 10 minutes at room temperature. Sections were blocked with 5% bovine serum albumin (BSA) for 30 minutes. After removing the blocking solution, primary antibody against Ki-67 (1:200 dilution) was applied, and sections were incubated overnight at 4 °C in a humidified chamber. The next day, slides were washed thoroughly with PBS, incubated with horseradish peroxidase (HRP)-conjugated secondary antibody for 1 hour at room temperature, and washed again with PBS. Color development was performed using freshly prepared 3,3′-diaminobenzidine (DAB) substrate for 1–3 minutes, monitored under a microscope, and stopped by rinsing with tap water. Nuclei were counterstained with hematoxylin for about 1 minute, followed by bluing in running water. Sections were dehydrated through a graded ethanol series, cleared in xylene, and mounted with neutral mounting medium.

### CCK-8 Assay

Cells were seeded in 24-well plates and transfected with an HITT overexpression plasmid using a non-liposomal transfection reagent. Twenty-four hours post-transfection, cells were treated with MDL-800 at various concentrations for 24 hours. The medium was then removed, and 100 μL of 1× CCK-8 reagent (ABclonal, China) was added to each well. After 30 minutes of incubation, absorbance was measured at 450 nm using a microplate reader.

### Xenograft Tumor Formation in Nude Mice

Female nude mice aged 4–5 weeks were used for subcutaneous xenograft and pharmacodynamic studies. HCT116 cells stably overexpressing HITT and control HCT116 cells were cultured to the logarithmic growth phase, resuspended in PBS, and adjusted to a concentration of 1 × 10^6^ cells/mL. Mice were randomly divided into four groups: Control; HITT overexpression; MDL-800; HITT + MDL-800, with at least 5 mice per group. Under sterile conditions, 100 μL of the corresponding cell suspension was injected subcutaneously into the back of each mouse. Tumor length and width were measured twice weekly, and tumor volume was calculated using the formula: volume = (length × width2)/2. When tumor volume reached approximately 200 mm³, mice in the two drug-treated groups began receiving intraperitoneal injections of MDL-800 (10 mg/kg per dose), while the two non-drug groups received an equal volume of solvent control. Tumor volume and body weight were monitored regularly.

### Statistical analysis

Statistical analysis was performed using GraphPad Prism (version 8). Pearson correlation was used to assess correlations, and scatter plots were generated using the bioinformatics.com.cn online tool. Data are presented as mean ± standard error of the mean (SEM) or standard deviation (SD), as indicated. Significance was determined using Student’s t-test, one-way ANOVA with Tukey’s post-hoc test, or two-way ANOVA, as appropriate. A *P-*value < 0.05 was considered statistically significant.

### Reporting summary

Further information on research design is available in the Nature Research Reporting Summary linked to this article.

## Supplementary information

Supplementary Information

Reporting summary

Original Data File

Original data

## Data Availability

Data reported in this paper will be shared by the lead contact upon request.
