# Supplementary material for: LncRNA HITT stabilizes SIRT6 to restrain H3/H4 acetylation and suppress colorectal cancer growth
Source: Cell Death Dis. 2026 Jul 27;17(1):901. doi: 10.1038/s41419-026-09107-z (PMC13691115; doi:10.1038/s41419-026-09107-z)

Figure 1

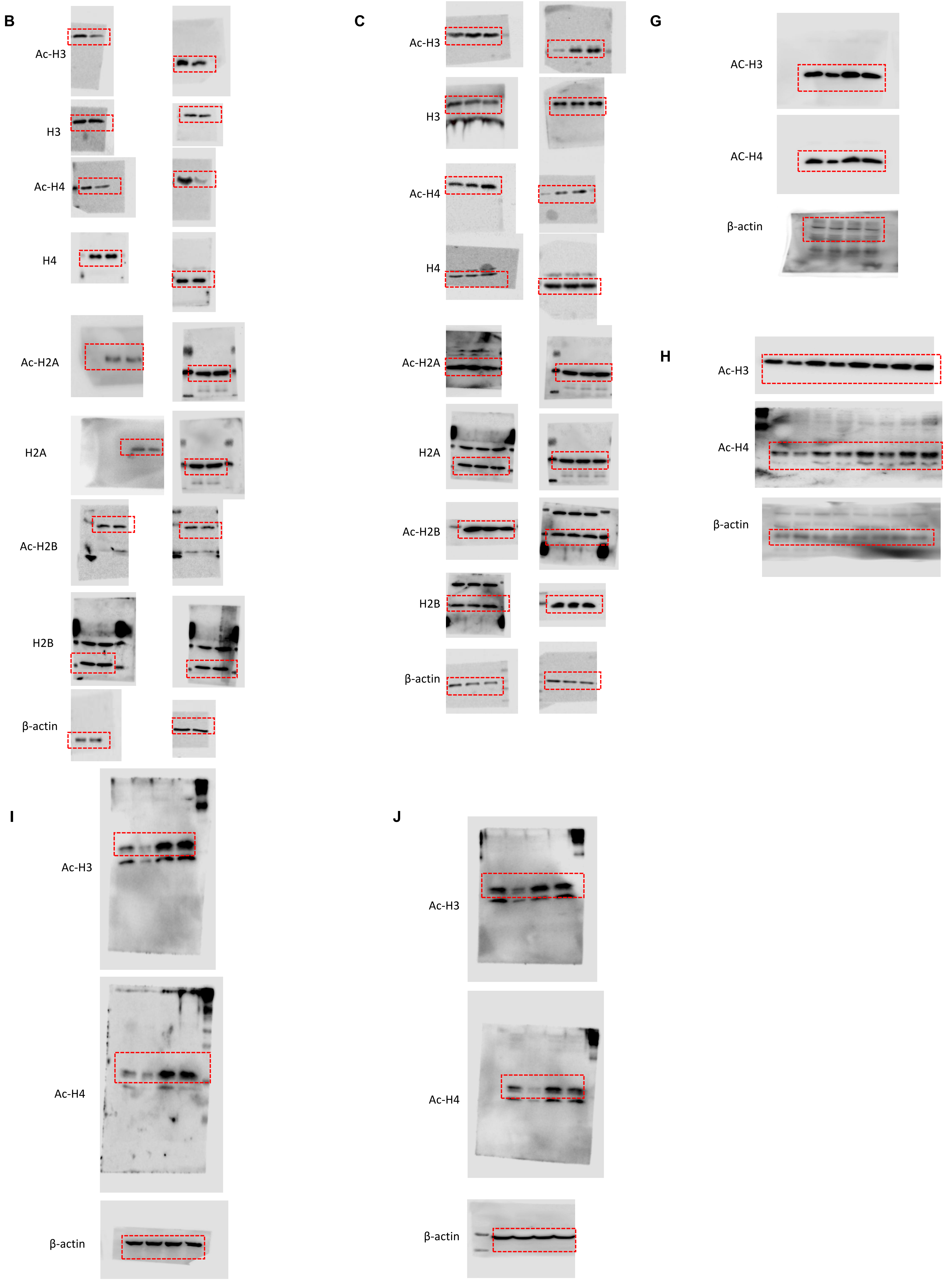
B
Ac-H3
H3
Ac-H4
H4
Ac-H2A
H2A
Ac-H2B
H2B
β-actin
C
Ac-H3
H3
Ac-H4
H4
Ac-H2A
H2A
Ac-H2B
H2B
β-actin
G
AC-H3
AC-H4
β-actin
H
Ac-H3
Ac-H4
β-actin
I
Ac-H3
Ac-H4
β-actin
J
Ac-H3
Ac-H4
β-actin

Figure S1

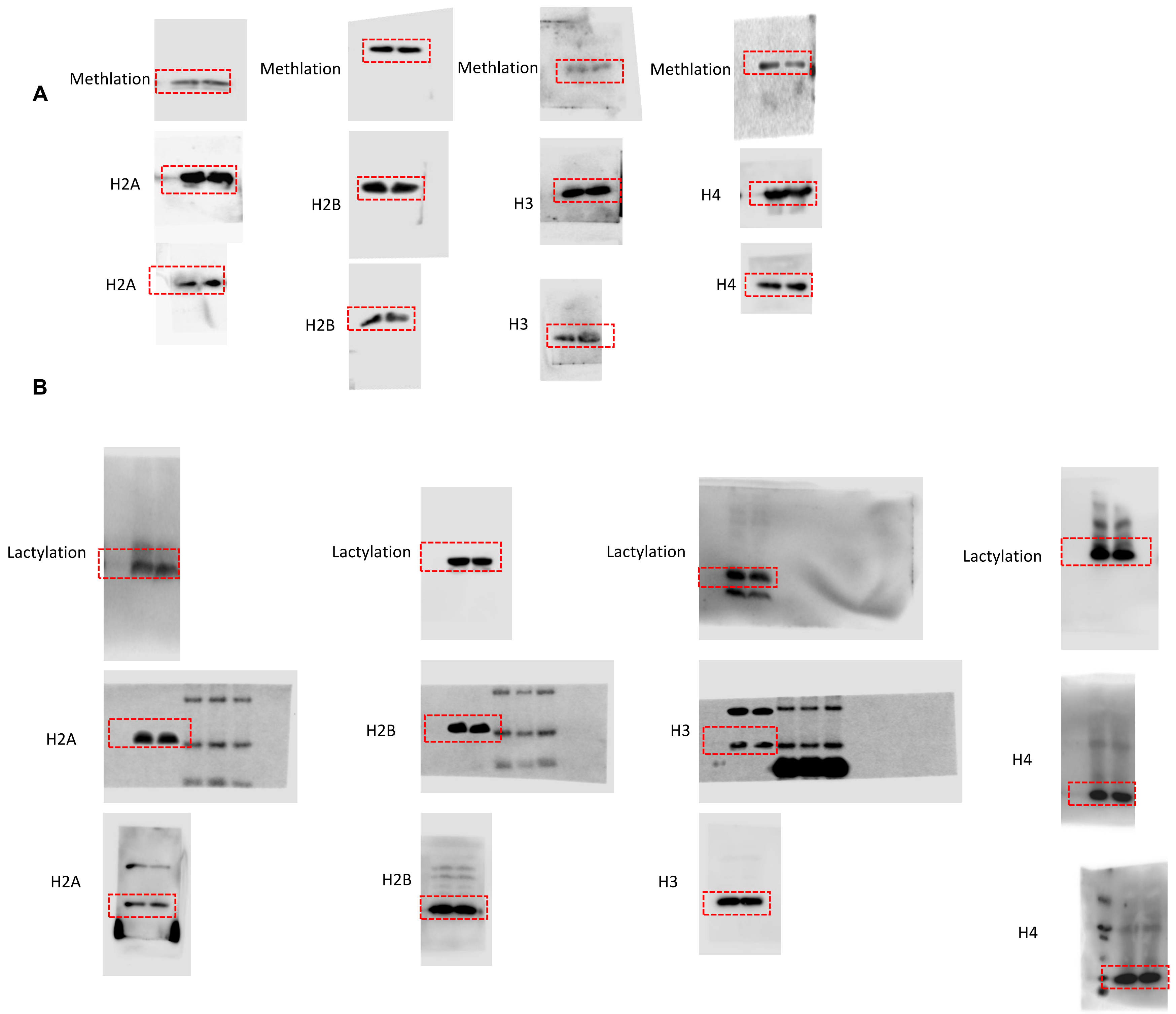
A
Methlation
Methlation
Methlation
Methlation
H2A
H2B
H3
H4
H2A
H2B
H3
H4
B
Lactylation
Lactylation
Lactylation
Lactylation
H2A
H2B
H3
H4
H2A
H2B
H3
H4

Figure S2

B

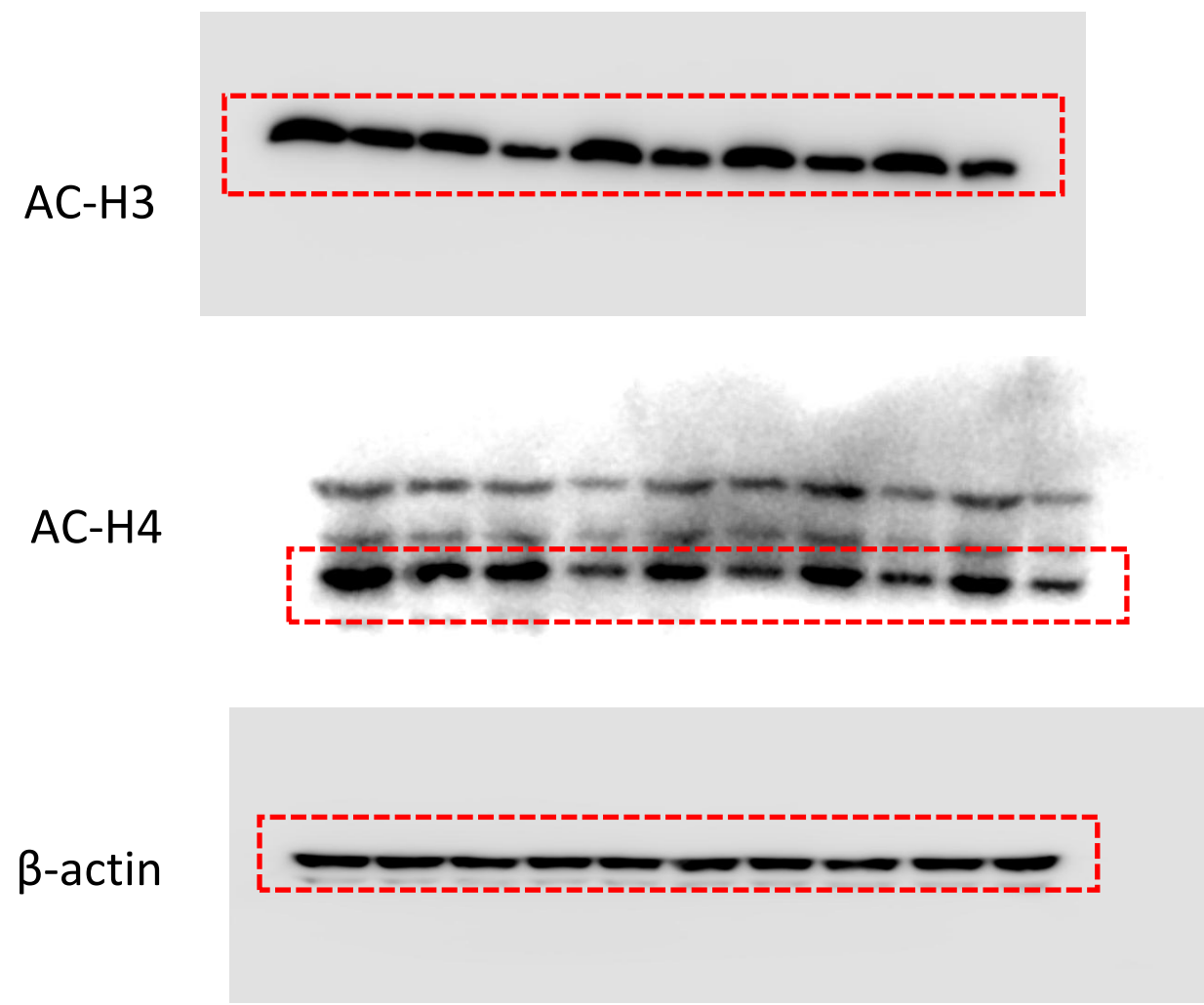

Figure 2

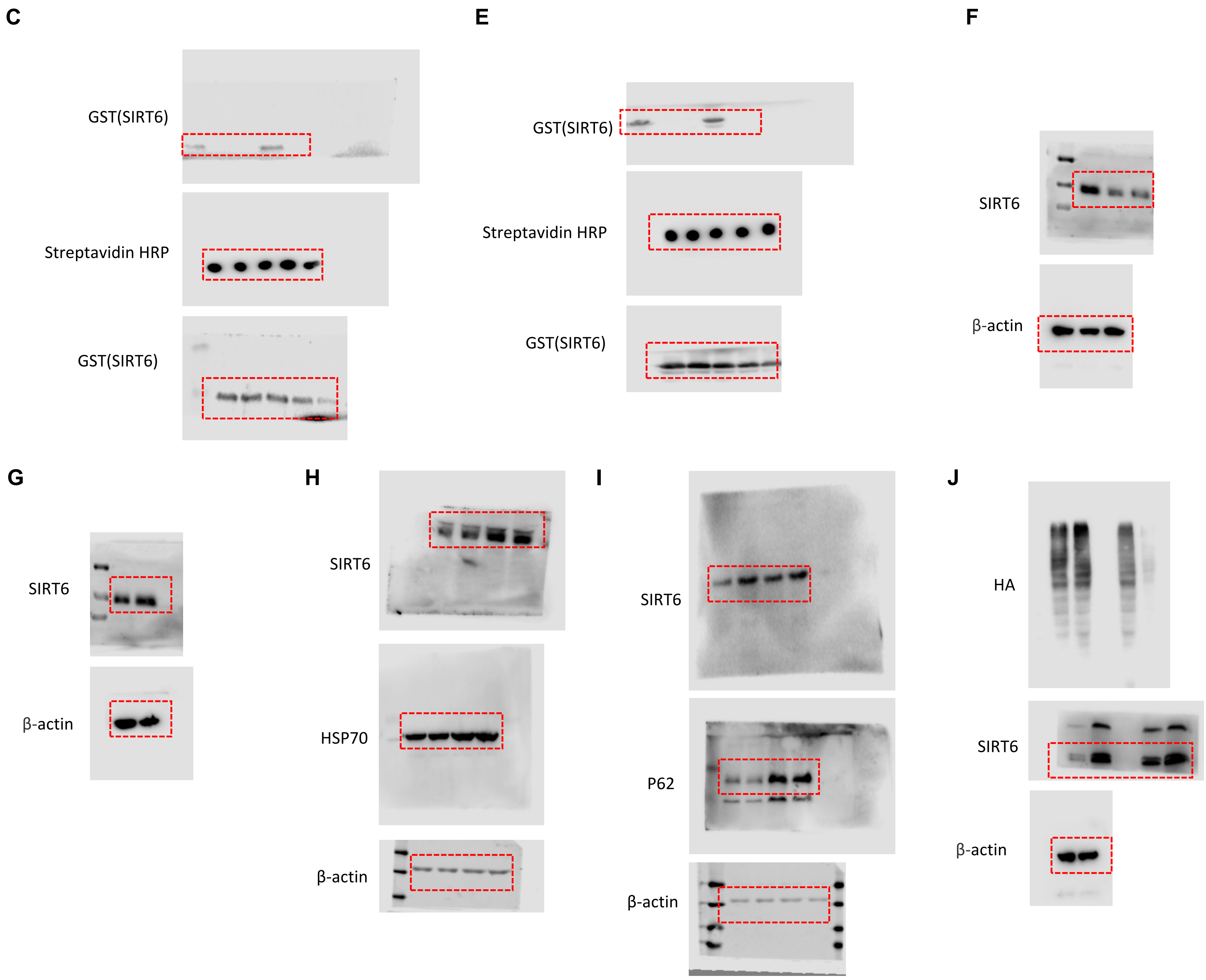

C
GST(SIRT6)
Streptavidin HRP
GST(SIRT6)
E
GST(SIRT6)
Streptavidin HRP
GST(SIRT6)
F
SIRT6
β-actin
G
SIRT6
β-actin
H
SIRT6
HSP70
β-actin
I
SIRT6
P62
β-actin
J
HA
SIRT6
β-actin

Figure 3

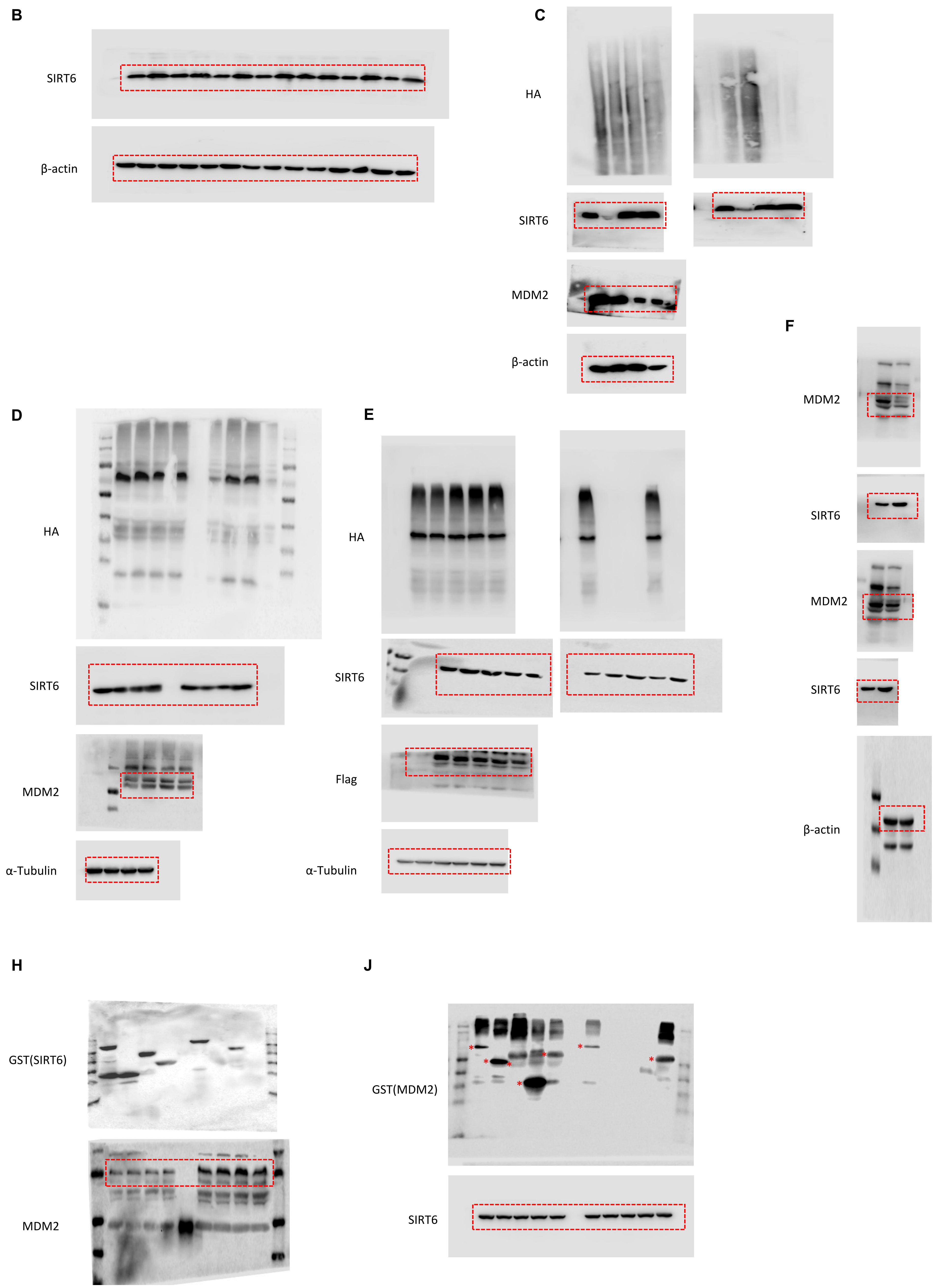
B
SIRT6
β-actin
C
HA
SIRT6
MDM2
β-actin
F
MDM2
SIRT6
MDM2
SIRT6
β-actin
D
HA
SIRT6
MDM2
α-Tubulin
E
HA
SIRT6
Flag
α-Tubulin
H
GST(SIRT6)
MDM2
J
GST(MDM2)
SIRT6

Figure S3

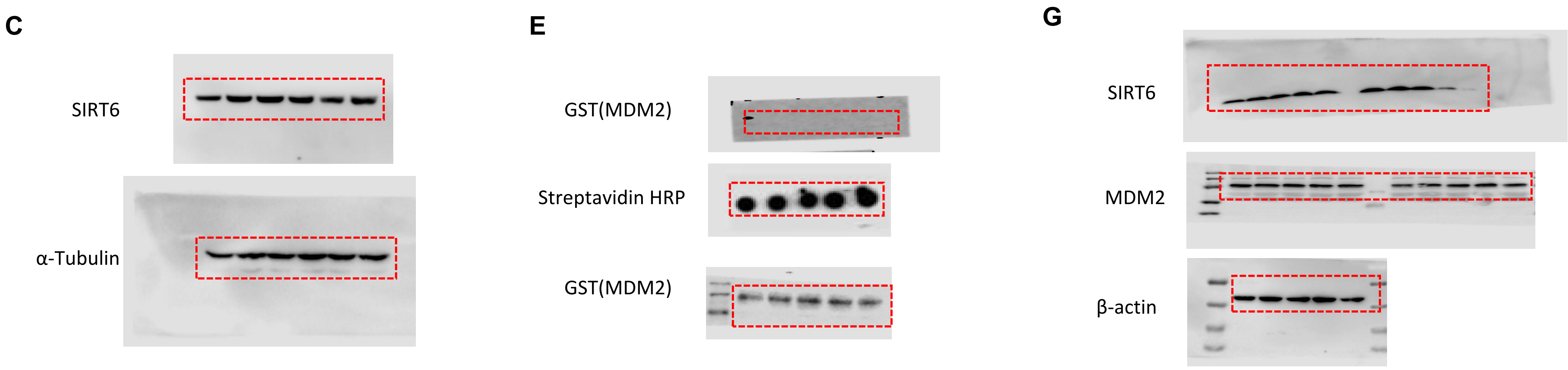
C
SIRT6
α-Tubulin
E
GST(MDM2)
Streptavidin HRP
GST(MDM2)
G
SIRT6
MDM2
β-actin

Figure 4

G

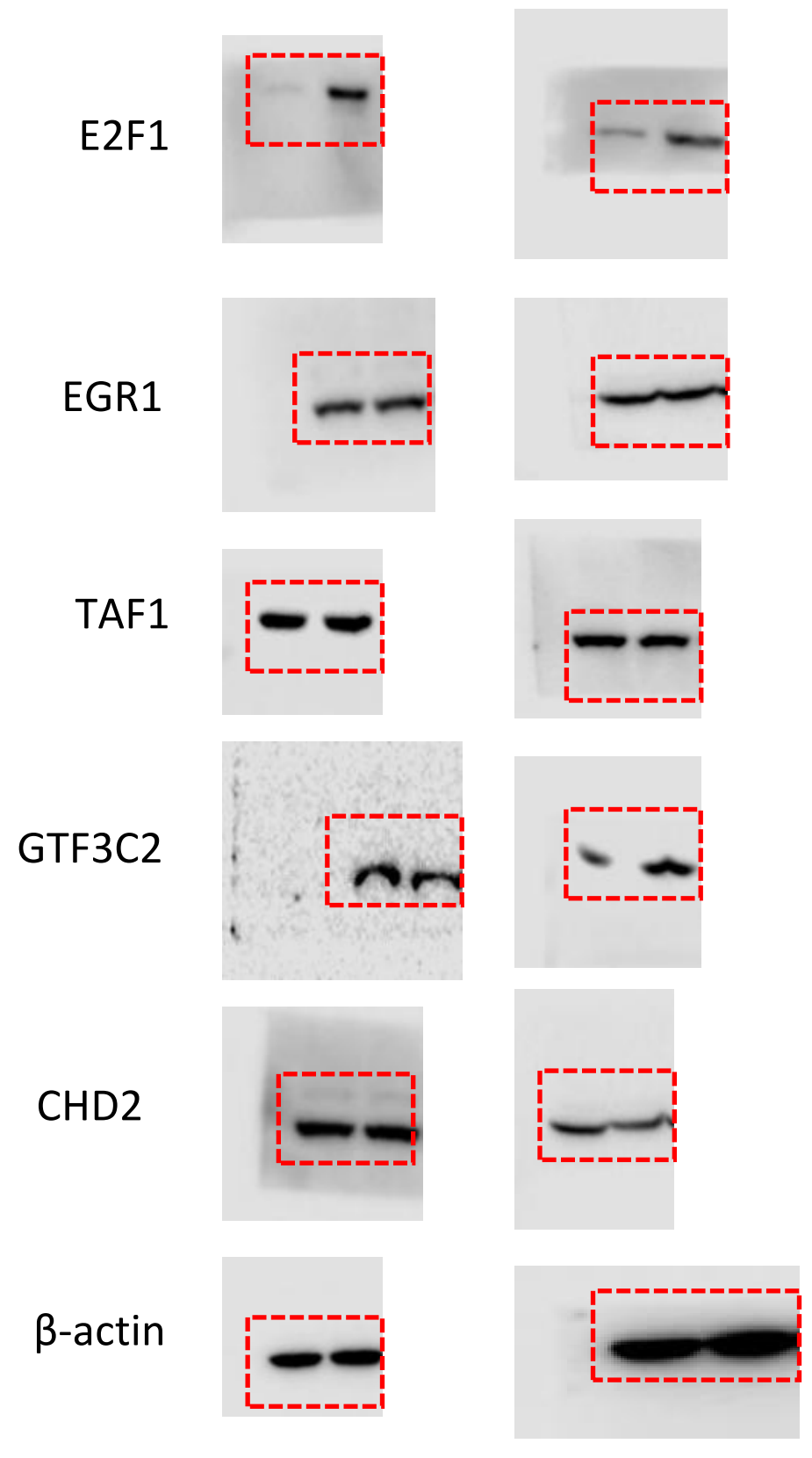

H

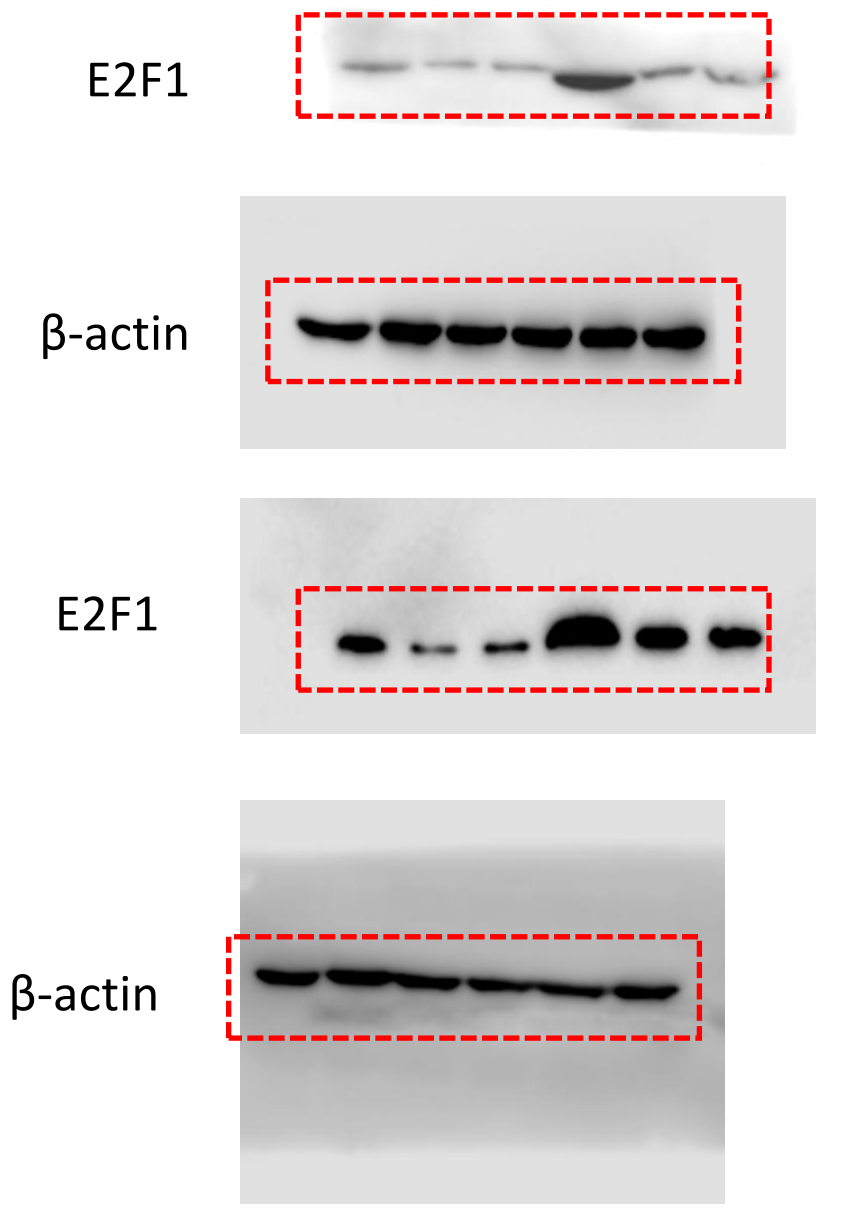

Figure S4

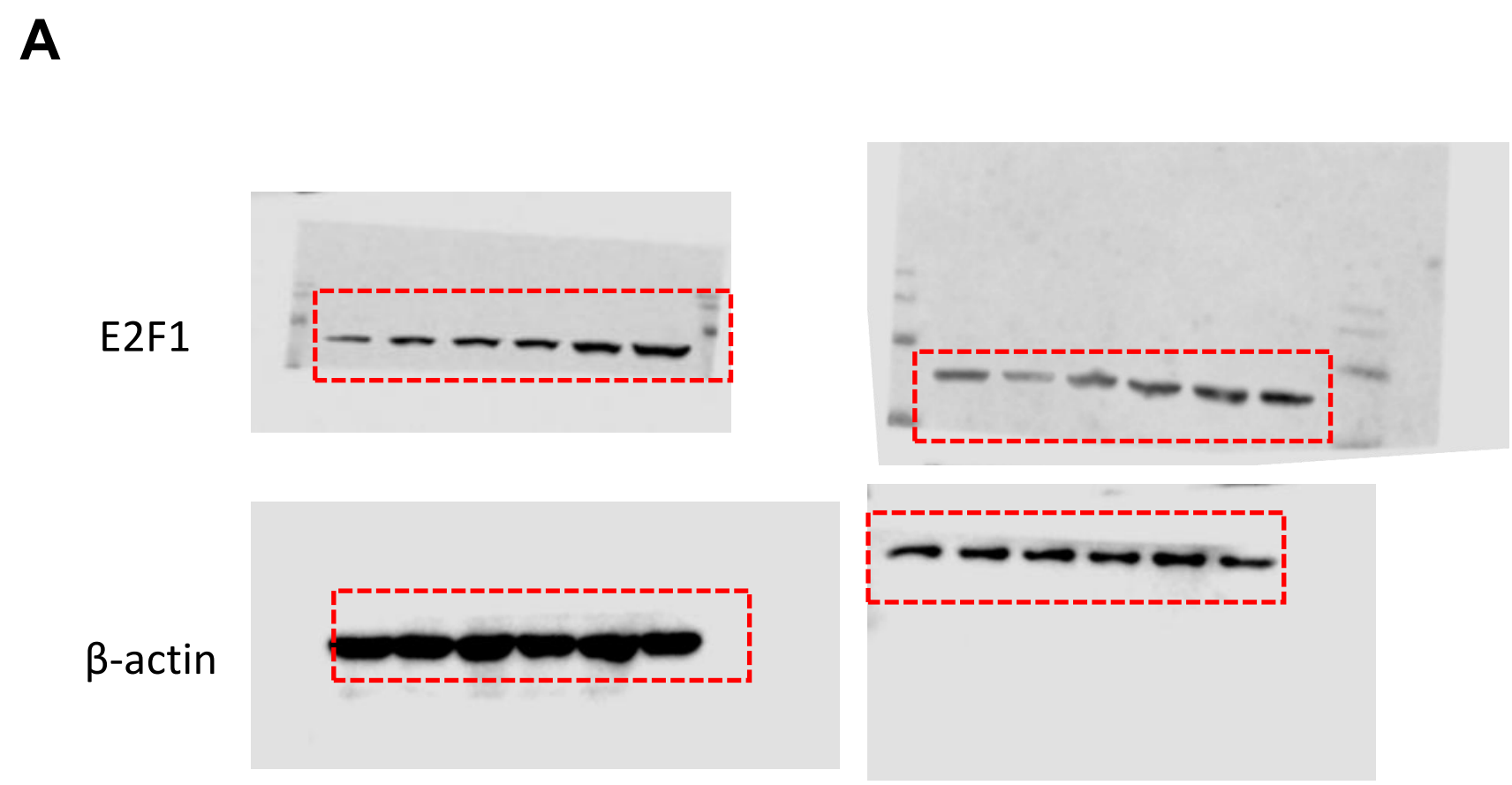

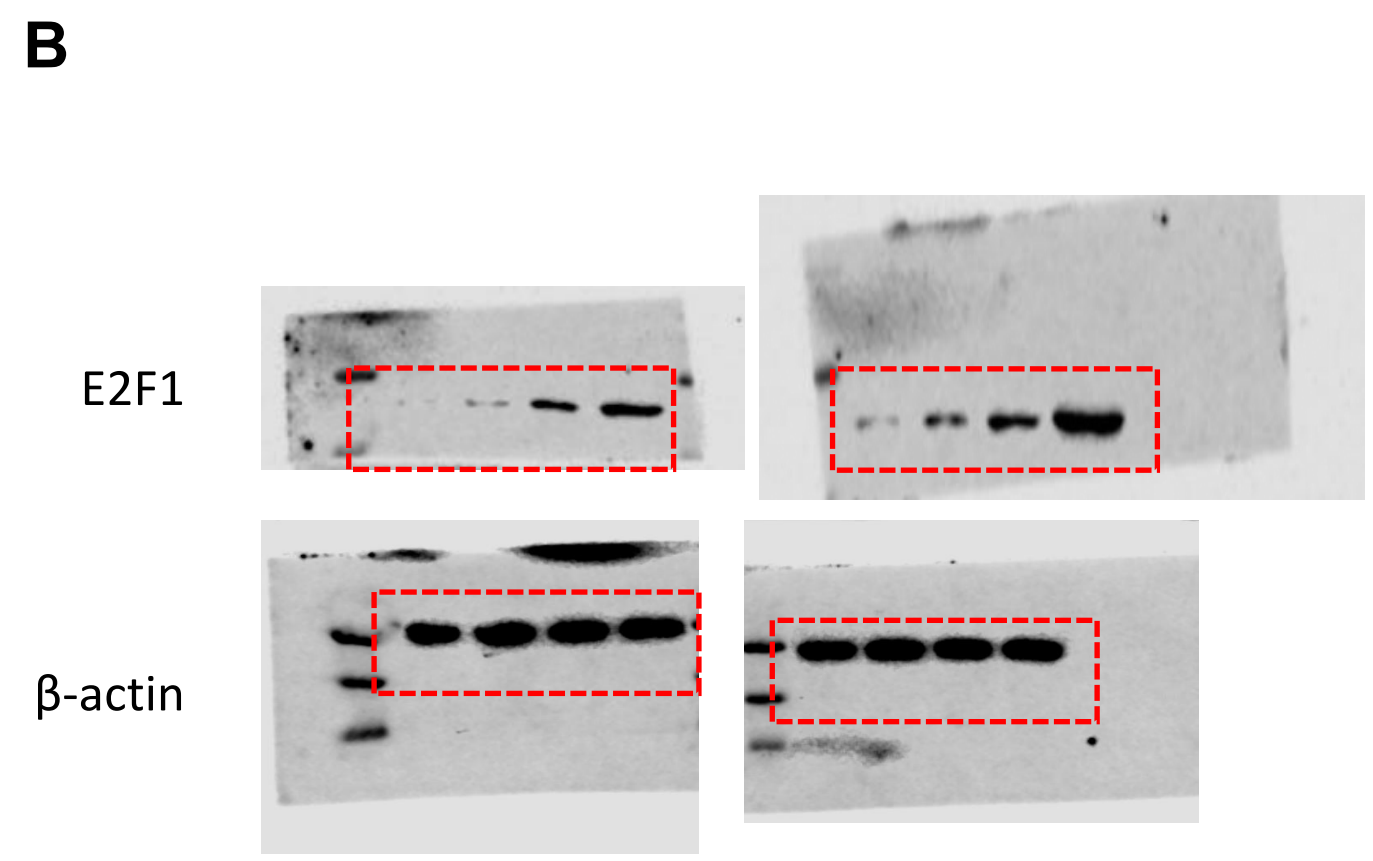

Figure S5

A

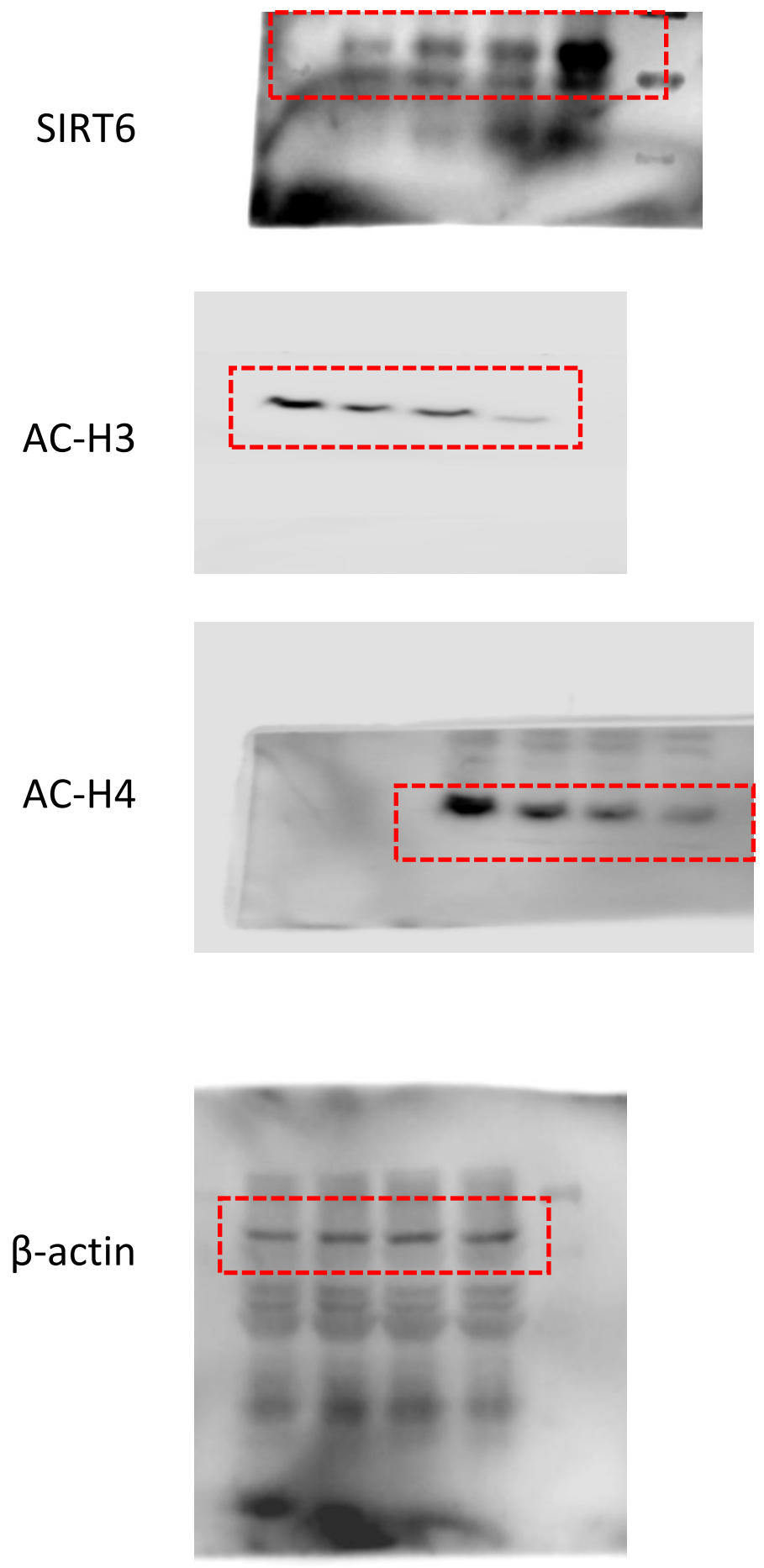

Supplement: Supplementary file 1 [file 41419_2026_9107_MOESM3_ESM.pdf]
