# Supplementary material for: LncRNA HITT stabilizes SIRT6 to restrain H3/H4 acetylation and suppress colorectal cancer growth
Source: Cell Death Dis. 2026 Jul 27;17(1):901. doi: 10.1038/s41419-026-09107-z (PMC13691115; doi:10.1038/s41419-026-09107-z)
Supplement: Supplementary file 2 [file 41419_2026_9107_MOESM1_ESM.docx]

**Supplementary Information for:**

**LncRNA HITT stabilizes SIRT6 to restrain H3/H4 acetylation and suppress colorectal cancer growth**

Bolun Li^1,#^, Zhiyuan Xiang^1,#^, Hao Liu^1,#^, Qiannan Cheng^1^, Haoyu Wang^1^, Kaiting Chen^1^, Jing Hu^1*^, Ying Hu^1,*^

^1^ Zhengzhou Advanced Research Institute, Harbin Institute of Technology, Zhengzhou 450000, Henan Province, China; School of Life Science and Technology, Harbin Institute of Technology, Harbin 150001, Heilongjiang Province, China; Key Laboratory of Science and Technology and Intelligent Equipment for the Prevention and Control of Major Chronic Diseases, Ministry of Industry and Information Technology, Harbin Institute of Technology, Harbin 150001, Heilongjiang Province, China.

^#^ These authors contributed equally.

^*^ Corresponding authors: Jing Hu, E-mail: jinghu@hit.edu.cn; Tel: +86-139-9799-6698; Ying Hu, Email: huying@hit.edu.cn; Tel: +86-451-86403826; Fax: +86-451-86403826.

**Contents:**

- Supplementary Figures S1–S5

- Supplementary Tables S1–S3

**Figure S1. HITT does not affect histone methylation or lactylation.**

(A, B) Representative Western blot images showing the methylation (A) and lactylation (B) levels of histones H2A, H2B, H3, and H4 in HITT-OE cells.

**Figure S2. HITT inhibits H3/H4 acetylation in an acetyltransferase-independent manner.**

(A) Venn diagram illustrating the overlap between H3 acetyltransferases and H4 acetyltransferases. (B) Representative Western blot images showing the acetylation levels of histones H3 and H4 in HITT-OE cells under knockdown of P300, CBP, GCN5L2, or TIP60.

**Figure S3. SIRT6 is a potential tumor suppressor, and HITT engages the deacetylase domain of SIRT6 without binding MDM2.**

(A) Box plot showing SIRT6 expression levels in colorectal cancer tissues compared with normal tissues. Statistical significance was determined by Student’s t-test *(P* < 0.001, Student's t-test). (B) Kaplan-Meier survival analysis of patients stratified by SIRT6 expression levels. (*P* = 0.0143, log-rank test). (C) Western blot analysis of SIRT6 protein levels in HCT116 cells with or without HITT overexpression, following treatment with MLN4924 (an inhibitor of Cullin-RING-type E3 ligases) or Heclin (a HECT-domain E3 ligase inhibitor). (D) Potential ubiquitination sites in SIRT6 predicted by the UbiBrowser database. Two lysine residues (K160 and K267) were identified as candidate sites. (E) *In vitro* RNA pull-down assay using biotin-labeled HITT followed by immunoblotting for MDM2. HITT showed no detectable binding to MDM2. (F) HCT116 cells were transfected with Flag-tagged full-length SIRT6 (FL) or truncation mutants corresponding to the N-terminal region (F1, amino acids 1–48), the deacetylase domain (F2, amino acids 49–271), or the C-terminal disordered region (F3, amino acids 272–355). CLIP was performed using anti-Flag antibody or control IgG, followed by qRT-PCR to quantify HITT enrichment. 18S rRNA served as a negative control. Data are shown as mean ± SEM (n = 3). (G) HCT116 cells were co-transfected with a fixed amount of Flag-MDM2 (5 μg) and increasing doses of HITT-overexpression plasmid (0, 1, 3, or 6 μg), or with Lnc00626 (5 μg) as an unrelated lncRNA control. Anti-Flag immunoprecipitation was performed at 48 h post-transfection, and co-immunoprecipitated endogenous SIRT6 was detected by Western blot (left). β-actin served as a loading control, and IgG was used as a non-specific IP control. HITT mRNA expression in the corresponding samples was quantified by qRT-PCR (right). Data are shown as mean ± SEM (n = 3). ***P* < 0.01, *****P* < 0.0001 by one-way ANOVA; N.S., not significant.

**Figure S4. MDL-800 induces the expression of E2F1**

(A) The expression level of E2F1 was determined by Western blots in HCT116 and LoVo cells after MDL-800 (0.1 mM) treatment for the indicated time periods. (B) The expression level of E2F1 was determined by Western blot in HCT116 and LoVo cells treated with different doses of MDL-800 for 24 h.

**Figure S5. SIRT6 stabilization and histone H3/H4 deacetylation are recapitulated in xenograft tumor tissues, and HITT overexpression efficiency is confirmed *in vivo*.**

Tumor tissues from the four treatment groups (Vector, HITT-OE, MDL-800, and HITT-OE + MDL-800; n = 5 mice per group) described in Fig. 5G were collected, homogenized, and subjected to protein and RNA extraction. (A) Representative Western blot images showing the protein levels of SIRT6 and the acetylation status of histones H3 (Ac-H3) and H4 (Ac-H4) in tumor tissues. β-actin served as the loading control. (B) qRT-PCR analysis of HITT mRNA expression in the corresponding tumor tissues, confirming sustained HITT overexpression *in vivo* and further induction of HITT by MDL-800 treatment. Data are shown as mean ± SEM (n = 5 biological replicates). ****P* < 0.001, *****P* < 0.0001 by one-way ANOVA with post-hoc test.

**Table S1 siRNA sequences used in this study.**

siRNA sequence (5’-3’)

| Gene | sequence |
| --- | --- |
| Control | UUUUCCGAACGUGUCACGUTT |
| *HITT*#1 | CCAGGAAGGCGAUUUACAATT |
| *HITT*#2 | CCUCAUGAAUGGGAUUAAUTT |
| *SIRT1* | GCACAGAUCCUCGAACAAUUC |
| *SIRT2* | CGCGUGCAGCGCGUUUCUUCUCC |
| *SIRT6* | AAGAAUGUGCCAAGUGUAAGA |
| *E2F1* | CGUGGACUCUUCGGAGAACTT |
| *MDM2* | AAGCCAUUGCUUUUGAAGUUA |
| *P300* | UCAAUAAUGCCUGGUUAUAUA |
| *CBP* | GUGACCGUAACCGUAUUUG |
| *GCN5L2* | AACCCCUGGGACAACAAGG |
| *TIP60* | AGAUGGUAGUGCUGGGGUUG |
| *SYVN1* | GCAUGGCAGUCCUGUACAUTT |
| *PML* | AUUACGUGACACGUUCGGAGAA |
| *BARD1* | AUUCAAAAAUGAUGGGCAAG |
| *LNX1* | GAGAACUCGAGUUCUCCUUG |
| *CBL* | GCCGAUGUGAAAUUAAAGGTA |

**Table S2 qRT-PCR primers**

Real-time RT-PCR primer (5’-3’)

| Gene | Forward | Reverse |
| --- | --- | --- |
| *HITT* | ACACAAATGCTGGCCTCTGTCA | GGCAAGTGGCAAAGCCTCTC |
| *SIRT6* | TCGACACCACCTTTGAGAGC | ACTGCGTCTTACACTTGGCA |
| *18S* | AACTTTCGATGGTAGTCGCCG | CCTTGGATGTGGTAGCCGTTT |

**Table S3 Antibody information**

| REAGENT | SOURCE | IDENTIFIER | DILUTION |
| --- | --- | --- | --- |
| Histone H3ac (pan-acetyl) | Thermo | PA5-114693 | 1:1000 |
| Histone H4ac (pan-acetyl) | Thermo | PA5-40084 | 1:1000 |
| Histone H2A.Zac (pan-acetyl) | Thermo | PA5-114690 | 1:1000 |
| H2Bac | Thermo | PA5-114691 | 1:1000 |
| Pan Methylated Lysine | Abbkine | ABM40195 | 1:500 |
| Anti-D-Lactyl Lysine | PTM BIO | PTM-1429RM | 1:1000 |
| Histone H2A.X | Proteintech | 68888-1-Ig | 1:2000 |
| Histone H2B | Proteintech | 15857-1-AP | 1:2000 |
| Histone H3 | Proteintech | 17168-1-AP | 1:2000 |
| Histone H4 | Proteintech | 16047-1-AP | 1:2000 |
| SIRT6 | Proteintech | 13572-1-AP | 1:2000 |
| MDM2 | Proteintech | 27883-1-AP | 1:2000 |
| GST | Proteintech | 10000-0-AP | 1:2000 |
| HA | Proteintech | 51064-2-AP | 1:2000 |
| P62 | Proteintech | 18420-1-AP | 1:2000 |
| HSP70 | Proteintech | 10995-1-AP | 1:2000 |
| E2F1 | Proteintech | 66515-1-Ig | 1:2000 |
| TAF1 | Proteintech | 20260-1-AP | 1:2000 |
| GTF3C2 | Proteintech | 27494-1-AP | 1:2000 |
| CHD2 | Proteintech | 21334-1-AP | 1:2000 |
| Ki-67 | Proteintech | 27309-1-AP | 1:2000 |
| Flag | Proteintech | 66008-4-Ig | 1:2000 |
| β-actin | Proteintech | 66009-1-Ig | 1:2000 |
| Goat anti-Rabbit IgG | Thermo | 31460 | 1:2000 |
| Goat anti-Mouse IgG | Thermo | 31430 | 1:2000 |
