# Supplementary material for: LncRNA HITT stabilizes SIRT6 to restrain H3/H4 acetylation and suppress colorectal cancer growth
Source: Cell Death Dis. 2026 Jul 27;17(1):901. doi: 10.1038/s41419-026-09107-z (PMC13691115; doi:10.1038/s41419-026-09107-z)
Supplement: Supplementary file 3 [file 41419_2026_9107_MOESM2_ESM.docx]

**SPRINGER** **NATURE**

**Corresponding Author Name:** Ying Hu and Jing Hu (Co-corresponding authors)

**Manuscript Number:** CDDIS-26-0675

**Reporting Summary**

*Springer Nature wishes to improve the reproducibility of the work that we publish. This checklist is used to ensure good reporting standards and to improve the reproducibility. Please respond completely to all questions relevant to your manuscript. For more information, please read the journal's Guide to Authors.*

**☑ Check here to confirm that the following information is available in the Material & Methods section:**

• The exact sample size (n) for each experimental group/condition, given as a number, not a range

• A description of the sample collection allowing the reader to understand whether the samples represent technical or biological replicates (including how many animals, litters, culture, etc.)

• A statement of how many times the experiment shown was replicated in the laboratory

• Definitions of statistical methods and measures: For small sample sizes (n<5) descriptive statistics are not appropriate, instead plot individual data points

○ Very common tests, such as *t-test*, simple χ² tests, Wilcoxon and Mann-Whitney tests, can be unambiguously identified by name only, but more complex techniques should be described in the methods section

○ Are tests one-sided or two-sided?

○ Are there adjustments for multiple comparisons?

○ Statistical test results, e.g., *P* values

○ Definition of 'center values' as median or mean

○ Definition of error bars as s.d. or s.e.m. or c.i.

*Please ensure that the answers to the following questions are reported in the manuscript itself. We encourage you to include a specific subsection in the methods section for statistics, reagents and animal models. Below, provide the page number or section and paragraph number.*

| **Statistics and general methods** | **Reported in section/paragraph or page #** |
| --- | --- |
| 1. How was the sample size chosen to ensure adequate power to detect a pre-specified effect size? (Give section/paragraph or page #) For animal studies, include a statement about sample size estimate even if no statistical methods were used. | Methods, 'Statistical Analysis' section. For *in vitro* experiments, sample sizes (n = 3 independent biological replicates) were chosen based on standard practice in the field, which provides sufficient power to detect biologically meaningful differences. For the xenograft study, n = 5 mice per group was determined based on previous published xenograft studies using similar tumor models, which has been shown to provide adequate power to detect tumor volume differences. |
| 2. Describe inclusion/exclusion criteria if samples or animals were excluded from the analysis. Were the criteria pre-established? (Give section/paragraph or page #) | No samples or animals were excluded from the analysis. All data points obtained were included. No pre-established exclusion criteria were applied. |
| 3. If a method of randomization was used to determine how samples/animals were allocated to experimental groups and processed, describe it. (Give section/paragraph or page #) For animal studies, include a statement about randomization even if no randomization was used. | Methods, 'Xenograft Tumor Formation in Nude Mice' section. Mice were randomly divided into four groups (Vector control, HITT-OE, MDL-800, HITT-OE + MDL-800) before tumor implantation, with at least 5 mice per group. For *in vitro* experiments, no formal randomization was applied as cells from a single batch were equally distributed to all experimental conditions. |
| 4. If the investigator was blinded to the group allocation during the experiment and/or when assessing the outcome, state the extent of blinding. (Give section/paragraph or page #) For animal studies, include a statement about blinding even if no blinding was done. | For the xenograft study, tumor measurements (length and width) were performed by an investigator blinded to group allocation. For *in vitro* assays, no formal blinding was applied as the experiments involved direct molecular manipulations of identified groups, and outcomes were assessed by quantitative readouts (Western blot densitometry, qRT-PCR) using ImageJ or instrument software, minimizing observer bias. |
| 5. For every figure, are statistical tests justified as appropriate? Do the data meet the assumptions of the tests (e.g., normal distribution)? Is there an estimate of variation within each group of data? Is the variance similar between the groups that are being statistically compared? (Give section/paragraph or page #) | Methods, 'Statistical Analysis' section, and individual figure legends. Statistical tests were chosen based on the experimental design: two-tailed Student's *t*-test for two-group comparisons, one-way ANOVA with Tukey's post-hoc correction for multi-group comparisons, and two-way ANOVA for tumor growth curves. Data normality was assumed based on standard practice in the field for *in vitro* experiments. Variation is reported as SEM for *in vitro* data and SD for *in vivo* data, as indicated in each figure legend. Variance was comparable across groups in all comparisons performed. |

| **Reagents** | **Reported in section/paragraph or page #** |
| --- | --- |
| 6. Report the source of antibodies (vendor and catalog number) | Supplementary Table S3 lists all primary antibodies used in this study, including vendor names, catalog numbers, and dilution ratios. |
| 7. Identify the source of cell lines and report if they were recently authenticated (e.g., by STR profiling) and tested for mycoplasma contamination | Methods, 'Cell Culture and Transfection' section. Human colorectal cancer cell lines HCT116 and LoVo were purchased from the American Type Culture Collection (ATCC). Cell line authentication was confirmed by the vendor through STR profiling at the time of purchase. All cell lines were routinely tested for mycoplasma contamination and confirmed to be negative before use in this study. |

| **Animal Models** | **Reported in section/paragraph or page #** |
| --- | --- |
| 8. Report species, strain, sex and age of animals | Methods, 'Xenograft Tumor Formation in Nude Mice' section. Female BALB/c nude mice aged 4–5 weeks were used for subcutaneous xenograft and pharmacodynamic studies. |
| 9. For experiments involving live vertebrates, include a statement of compliance with ethical regulations and identify the committee(s) approving the experiments. | 'Ethics' section. All animal procedures were performed in accordance with the relevant guidelines and regulations, following the Rules for Animal Experiments published by the Chinese Government (Beijing, China). All experimental protocols were approved by the Research Ethics Committee of Harbin Institute of Technology, China (approval number: IACUC-2020002). |

*10. We recommend consulting the ARRIVE guidelines (PLoS Biol. 8(6), e1000412, 2010) to ensure that other relevant aspects of animal studies are adequately reported.*

Consulted. The ARRIVE guidelines were consulted in the design and reporting of the animal study.

| **Human subjects** | **Reported in section/paragraph or page #** |
| --- | --- |
| 11. Identify the committee(s) approving the study protocol. | n/a. This study did not involve human subjects. Patient survival and gene expression data were obtained from the publicly available TCGA-COAD dataset, which is fully de-identified. |
| 12. Include a statement confirming that informed consent was obtained from all subjects. | n/a. This study did not involve human subjects requiring informed consent. |
| 13. For publication of patient photos, include a statement confirming that consent to publish was obtained. | n/a. No patient photos were used in this study. |
| 14. Report the clinical trial registration number (at ClinicalTrials.gov or equivalent). | n/a. This study is not a clinical trial. |

*15. For phase II and III randomized controlled trials, please refer to the CONSORT statement and submit the CONSORT checklist with your submission.*

n/a.

*16. For tumor marker prognostic studies, we recommend that you follow the REMARK reporting guidelines.*

n/a. This study is a mechanistic investigation; the survival analysis (Fig. S3B) was based on the publicly available TCGA-COAD dataset rather than an original clinical cohort, and tumor marker prognostic evaluation was not the primary aim of this study.

| **Data deposition** | **Reported in section/paragraph or page #** |
| --- | --- |
| 17. Provide accession codes for deposited data. Data deposition in a public repository is mandatory for: a. Protein, DNA and RNA sequences b. Macromolecular structures c. Crystallographic data for small molecules d. Microarray data | n/a. No new sequencing data, structural data, or microarray data were generated in this study. Public TCGA-COAD data used for bioinformatics analysis are accessible through the TCGA portal (https://portal.gdc.cancer.gov/). All data supporting the findings of this study are available within the article and its Supplementary Information files. Original Western blot images and other source data will be provided by the lead contact upon reasonable request, as stated in the 'Data availability' section. |

*Deposition is strongly recommended for many other datasets for which structured public repositories exist; more details on our data policy are available in the Guide to Authors. We encourage the provision of other source data in supplementary information or in unstructured repositories such as Figshare and Dryad. We encourage publication of Data Descriptors (see Scientific Data) to maximize data reuse.*

| 18. If computer code was used to generate results that are central to the paper's conclusions, include a statement in the Methods section under 'Code availability' to indicate whether and how the code can be accessed. Include version information as necessary and any restrictions on availability. | n/a. No custom computer code was generated for this study. All bioinformatics analyses were performed using publicly available tools (UbiBrowser: http://ubibrowser.bio-it.cn; UCSC Genome Browser: https://genome.ucsc.edu; AlphaFold v3 for structural modeling) and standard software (GraphPad Prism v8 for statistical analysis; ImageJ for densitometric quantification of Western blots). |
| --- | --- |

*Academic Journals Reporting Checklist, November 2020*
